# Caspase-8 loss radiosensitizes head and neck squamous cell carcinoma to SMAC mimetic–induced necroptosis

**DOI:** 10.1172/jci.insight.139837

**Published:** 2020-12-03

**Authors:** Burak Uzunparmak, Meng Gao, Antje Lindemann, Kelly Erikson, Li Wang, Eric Lin, Steven J. Frank, Frederico O. Gleber-Netto, Mei Zhao, Heath D. Skinner, Jared Newton, Andrew G. Sikora, Jeffrey N. Myers, Curtis R. Pickering

**Affiliations:** 1 Department of Head and Neck Surgery, The University of Texas MD Anderson Cancer Center, Houston, Texas, USA.; 2 MD Anderson UTHealth Graduate School of Biomedical Sciences, Houston, Texas USA.; 3 Department of Radiation Oncology, The University of Texas MD Anderson Cancer Center, Houston, Texas, USA.; 4 Department of Radiation Oncology, University of Pittsburgh Hillman Cancer Center, Pittsburgh, Pennsylvania, USA.; 5 Bobby R. Alford Department of Otolaryngology - Head and Neck Surgery, Baylor College of Medicine, Houston, Texas, USA.

**Keywords:** Cell Biology, Oncology, Apoptosis pathways, Head and neck cancer, Radiation therapy

## Abstract

Caspase-8 (*CASP8*) is one of the most frequently mutated genes in head and neck squamous carcinomas (HNSCCs), and *CASP8* mutations are associated with poor survival. The distribution of these mutations in HNSCCs suggests that they are likely to be inactivating. Inhibition of CASP8 has been reported to sensitize cancer cells to necroptosis, a regulated cell death mechanism. Here, we show that knockdown of CASP8 renders HNSCCs susceptible to necroptosis by a second mitochondria-derived activator of caspase (SMAC) mimetic, birinapant, in combination with pan-caspase inhibitors Z-VAD-FMK or emricasan and radiation. In a syngeneic mouse model of oral cancer, birinapant, particularly when combined with radiation, delayed tumor growth and enhanced survival under CASP8 loss. Exploration of molecular underpinnings of necroptosis sensitivity confirmed that the level of functional receptor-interacting serine/threonine protein kinase 3 (RIP3) determines susceptibility to this mode of death. Although an in vitro screen revealed that low RIP3 levels rendered many HNSCC cell lines resistant to necroptosis, patient tumors maintained RIP3 expression and should therefore remain sensitive. Collectively, these results suggest that targeting the necroptosis pathway with SMAC mimetics, especially in combination with radiation, may be relevant therapeutically in HNSCC with compromised CASP8 status, provided that RIP3 function is maintained.

## Abstract 2

Caspase-8 status sensitizes a murine model of head and neck squamous carcinoma to the combination of radiation and SMAC mimetic treatment through increased necroptosis.

## Introduction

Head and neck squamous carcinoma (HNSCC), which comprises epithelial tumors originating from the mucosa of oral cavity, oropharynx, larynx, and hypopharynx, is one of the most common cancers in the world, with the diagnosis of nearly 650,000 new cases and more than 300,000 cancer-related deaths annually (1). The 5-year survival rate for HNSCC remains at approximately 50% because of resistance to standard-of-care therapy that involves surgery, radiation and/or platinum- or taxane-based chemotherapy, or combination of these modalities (2). Integrative genomic analysis of HNSCC has uncovered that caspase-8 (*CASP8*) is one of the most frequently mutated genes in HNSCC, with somatic mutations detected in approximately 10% of cases (3, 4). The distribution of *CASP8* mutations observed in patient tumors and cell lines suggests that they are likely to be inactivating-type mutations where protein function is compromised (4).

CASP8 is an aspartate-specific cysteine protease that plays a key role in the initiation of extrinsic apoptosis (5). Binding of a death ligand (i.e., TNF-related apoptosis-inducing ligand, TRAIL) to its cognate receptor (i.e., TRAIL receptor) leads to formation of a death-inducing signaling complex (DISC) at the cytoplasmic tail of the death receptor that comprises the adapter protein FADD (Fas-associated with death domain) and procaspase-8. Processing of procaspase-8 within the DISC yields active CASP8, which translocates to the cytosol to cleave and activate its downstream executioner caspases such as caspase-3 and caspase-7, executing the apoptosis pathway (6–8). Because of the key role it plays in death receptor–mediated apoptosis, CASP8 has long been considered a tumor suppressor gene (9). This is consistent with the observation that CASP8 activity is impaired in a variety of cancer types, such as neuroblastoma, medulloblastoma, and HNSCC, through mutations and epigenetic silencing (4, 10, 11). However, the presence of functional CASP8 is also crucial for the maintenance of life because *Casp8^−/−^* mice die intranatally around embryonic day 11, resulting from uncontrolled necroptosis (12).

Necroptosis is a unique mechanism of regulated cell death stimulated upon death receptor signaling (i.e., TNFA signaling) that relies on the activation of mixed lineage kinase domain-like (MLKL), a pseudokinase, by receptor-interacting serine/threonine protein kinases 1 and 3 (RIP1 and RIP3). CASP8 regulates kinase activity of RIP1 and RIP3, both of which contain CASP8 cleavage sites (13, 14). TNFA binding to its cognate receptor, TNFR1, leads to formation of complex 1 that contains TNFR-associated death domain (TRADD), TNFR-associated factor 2 (TRAF2), inhibitor of apoptosis proteins (IAPs) cIAP1/cIAP2, and RIP1. Ubiquitylation of RIP1 by cIAP1/cIAP2 within complex 1 culminates in the activation of the canonical NF-κB pathway. When cIAPs are inhibited pharmacologically, such as with the second mitochondria-derived activator of caspase (SMAC) mimetic birinapant, RIP1 recruits CASP8 to form cytosolic complex 2 to initiate apoptosis (15). In cases where CASP8 is inhibited by chemicals, such as Z-VAD-FMK (carbobenzoxy-valyl-alanyl-aspartyl-[O-methyl]-fluoromethylketone), CASP8 regulation over RIP1/RIP3 kinase activity is abrogated, which results in the assembly of complex 2C (also referred to as necrosome) in the cytosol, consisting of RIP1, RIP3, and MLKL (16). MLKL is phosphorylated, trimerized, and activated within complex 2C, upon which it translocates to the plasma membrane to induce membrane permeabilization and subsequent necroptotic cell death (17).

SMAC mimetics are small-molecule inhibitors that promote caspase activation and apoptosis through neutralization of IAPs (18). Preclinical studies have highlighted the therapeutic potential of SMAC mimetics through induction of cancer cell death directly (19) or via synergistic interaction with a variety of cytotoxic therapy approaches, including chemotherapy (20, 21), radiotherapy (22, 23), or immunotherapy (24). The SMAC mimetic birinapant was found to enhance cytotoxicity induced by death ligands in a panel of HNSCC cell lines (25). Birinapant also synergizes with radiation to prevent tumor growth in various xenograft models of HNSCC bearing genomic amplifications of FADD and cIAP1 in vivo (25). Other SMAC mimetic compounds such as LCL161 and ASTX660 have also been shown to confer in vivo radiosensitivity to HNSCC xenografts (26, 27). However, how mutations and/or loss of *CASP8* affects necroptosis in HNSCC and whether modulation of the necroptosis pathway with these small-molecule agents might have potential clinical utility in the context of *CASP8* loss have largely been unexplored.

In this study, we found that deletion of *CASP8* rendered HNSCCs susceptible to necroptosis induced by the SMAC mimetic birinapant. Inhibition of CASP8 function was also associated with enhanced necroptotic killing by radiation when combined with birinapant in vitro and in vivo. We further demonstrated that the level of RIP3 expression determines necroptosis sensitivity in HNSCC. These findings provide preclinical justification for use of the necroptosis pathway as a therapeutic target in patients with HNSCC.

## Results

### CASP8 mutations are associated with radioresistance and poor survival outcomes in HNSCC.

**Figure 1:**
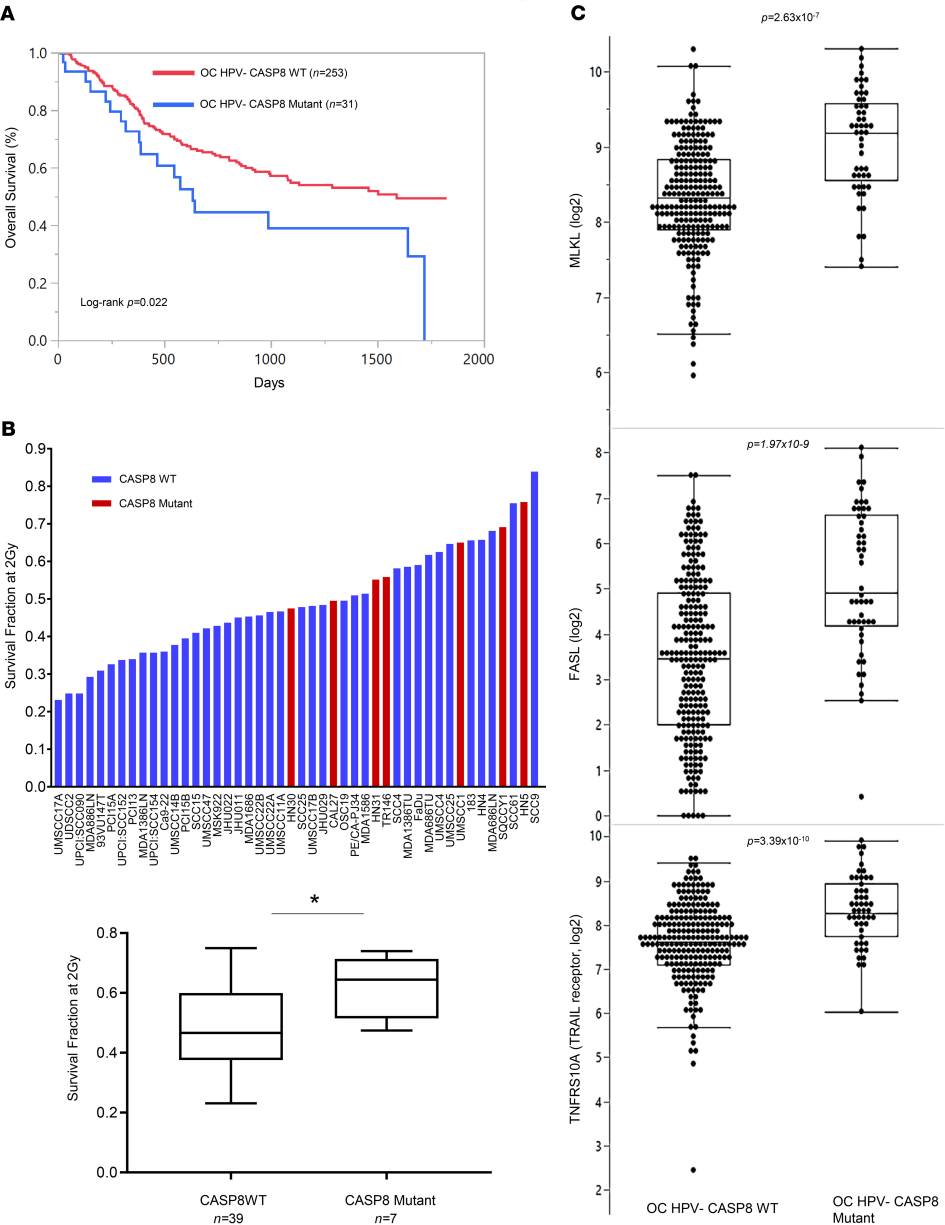
*CASP8* mutations are associated with radioresistance and poor survival outcomes in HNSCCs. (**A**) Kaplan-Meier survival plots for *CASP8* in 284 HPV-negative (HPV–) oral cavity (OC) HNSCCs in TCGA. Red line represents HPV-negative CASP8 WT or Hras proto-oncogene, GTPase–mutant (HRAS-mutant) OC cases; *n* = 253. Blue line represents HPV-negative CASP8-mutant, HRAS WT OC cases; *n* = 31. Please refer to Supplemental Table 1 for the multivariate analysis. (**B**) A panel of 46 HNSCC cell lines were sequenced for *CASP8* and evaluated for sensitivity to radiation using clonogenic survival assays. Surviving fraction following 2 Gy of ionizing radiation (XRT) was used to determine radiosensitivity. Cell lines with *CASP8* mutation are marked in red. HN5 and TR146 mutations are missense, while all others are nonsense, frameshift, or splice site. Cell lines were then grouped for *CASP8* status, and the average clonogenic survival data are shown for each group. Two-tailed Student’s *t* test was used for statistics. *P < 0.05. (**C**) Scatter plots show gene expression for MLKL, FASL, and TNFRSF10A (TRAIL receptor) by *CASP8* status in TCGA HPV-negative oral cancer samples. Mean values are shown by the bar. *P* values were computed using Mann-Whitney *U* test.

Alterations of *CASP8*, most of which are mutations, are found frequently (11.2%) in HNSCCs (4) and are associated with poor overall survival on univariate (Figure 1A) and multivariate analysis (Supplemental Table 1; supplemental material available online with this article; https://doi.org/10.1172/jci.insight.139837DS1). To determine whether poor overall survival outcomes might be linked to radioresistance in *CASP8*-mutant HNSCCs, we selected a panel of 46 HNSCC cell lines with known *CASP8* status (28). We examined sensitivity to increasing doses of radiation using a standard clonogenic survival assay (29) and determined the level of radiosensitivity using survival fraction at the 2 Gy dose, since 2 Gy is the dose typically used in clinical practice to treat patients with HNSCC. Interestingly, we found that *CASP8*-mutant cell lines were significantly more radioresistant than those with WT *CASP8* (Figure 1B) (4). Since CASP8 is known to regulate necroptosis and necroptosis can be induced by radiation in a variety of cancers (30, 31), we aimed to understand whether necroptosis can be exploited to radiosensitize *CASP8*-mutant HNSCCs to radiation. We first explored whether necroptosis-related genes are differentially expressed between *CASP8*-mutant and WT tumors in The Cancer Genome Atlas (TCGA). The top differentially expressed genes included at least 3 necroptosis-related genes. *MLKL* (*P* = 3.39 × 10^–10^), Fas ligand (*FASLG*, *P* = 1.97 × 10^–9^), and TNF receptor superfamily member 10A, TRAIL receptor, DR4 (*TNFRSF10A*, *P* = 2.63 × 10^–7^) showed higher expression in *CASP8*-mutant HPV-negative oral cancers than their WT counterparts, suggesting that necroptosis might be a potential pathway to target in *CASP8*-mutant HNSCCs (Figure 1C).

### Knockdown of CASP8 sensitizes HNSCCs to necroptotic death by birinapant and Z-VAD-FMK.

**Figure 2:**
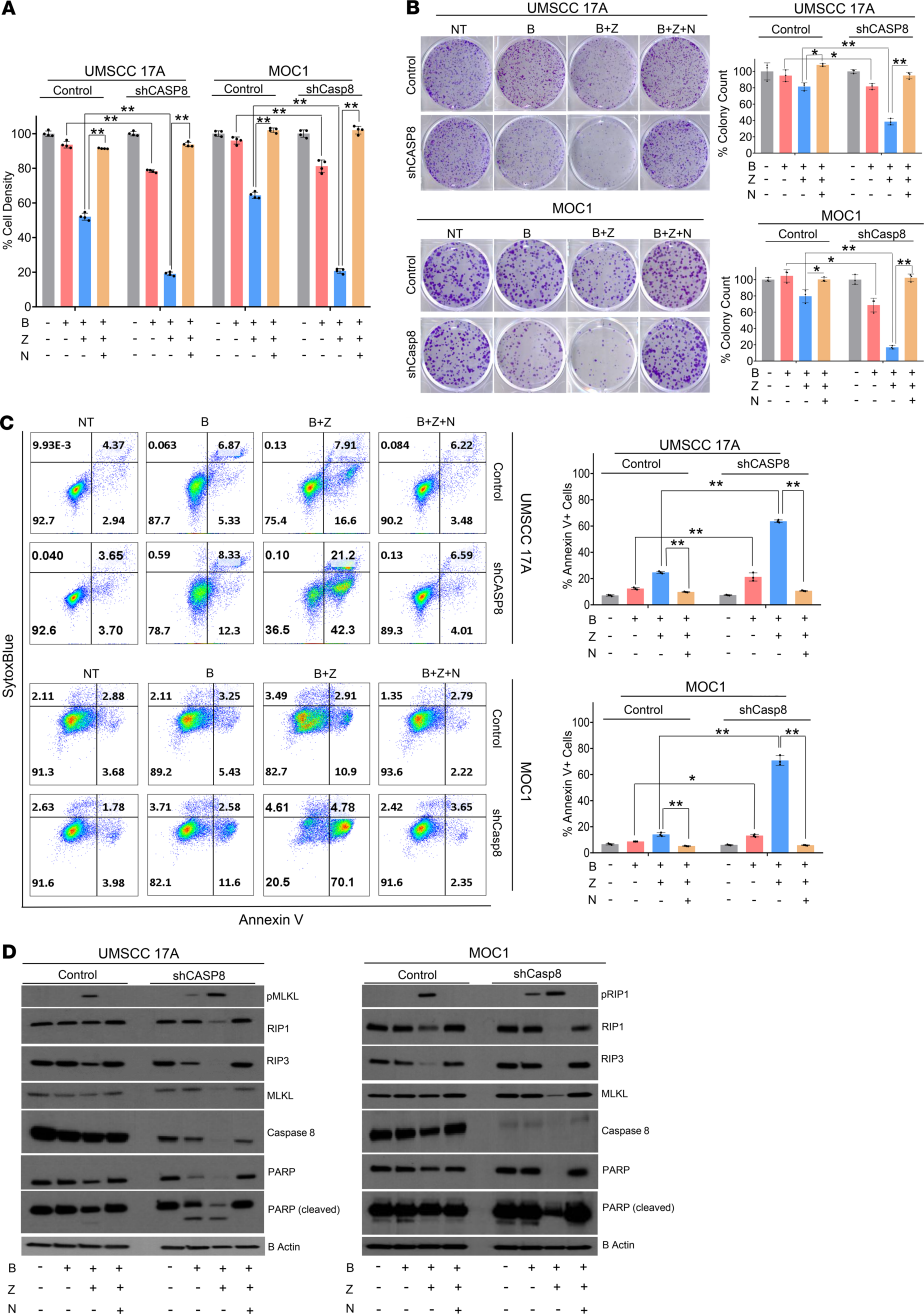
Knockdown of CASP8 sensitizes HNSCCs to necroptotic death by birinapant and Z-VAD-FMK. UMSCC 17A and MOC1 control and shRNA knockdown (shCASP8) cells were treated with birinapant (B [200 nmol/L for the UMSCC 17A cells; 1 μmol/L for the MOC1 cells]), Z-VAD-FMK (Z [5 μmol/L for both the cell lines]), necrostatin-1s (N [10 μmol/L for both the cell lines]), or the combinations as indicated for 24 hours. (**A**) Cell viability was assessed using CellTiter-Glo. Values normalized to nontreated cells from the same experiment to calculate percentage of cell density. All treatments were carried out in replicates of 4. More detailed analysis can be found in Supplemental Figure 2, A and B, and Supplemental Figure 3. (**B**) Representative images of clonogenic survival assays. Twenty-four hours after treatments (birinapant doses reduced to 50 nmol/L and 250 nmol/L for the UMSCC 17A and MOC1 cells respectively, for this assay, and other drugs used at the same concentrations as stated above), drug dilutions were washed out, and colonies were allowed to form for 5–12 days, after which they were stained and counted. Surviving colonies were normalized to nontreated cells from the same experiment to calculate percentage of colony count. All treatments were carried out in triplicates. Please refer to Supplemental Figure 4 for more detailed version. (**C**) Annexin V-APC/SytoxBlue staining was performed 24 hours after treatments. Percentage of annexin V positivity was used as a measure to assess cell death. All treatments were carried out in triplicates. More detailed analysis can be found in Supplemental Figure 5. (**D**) Whole-cell lysates were collected 24 hours after treatments and subjected to Western blot analysis for the indicated key cell death markers; β-actin was used as loading control. One-way ANOVA with post hoc Bonferroni-corrected *t* test was used for statistics. *P < 0.05, **P < 0.001 for the indicated pairwise comparisons. All experiments detailed above were repeated 3 times with similar results. B, birinapant; NT, no treatment; X, radiation; Z, Z-VAD-FMK.

Inhibition or mutation of *CASP8* predisposes a wide variety of cancers to necroptosis (32). In an effort to understand how inactivating mutations of *CASP8* that result in loss of protein function affect necroptosis sensitivity in HNSCC, we stably knocked down CASP8 using a short hairpin RNA (shRNA) in 2 *CASP8* WT HNSCC cell lines, namely the human-derived UMSCC 17A cells and mouse-derived syngeneic oral cancer MOC1 HNSCCs (33). Knockdown of CASP8 alone did not significantly affect proliferative or colony-forming abilities of the cell lines (Supplemental Figure 1). The UMSCC 17A and MOC1 CASP8-knockdown cells and scrambled shRNA control cells (cells transduced with a lentiviral construct lacking the shRNA) were treated with the SMAC mimetic birinapant alone or in combination with the pan-caspase inhibitor Z-VAD-FMK at concentrations previously shown to be active (25). This combination is a standard method to experimentally induce necroptotic death (34). Cell survival was assessed by cell viability and clonogenic assays. Knockdown of CASP8 significantly increased the sensitivity of UMSCC 17A and MOC1 cells to single-agent birinapant and birinapant in combination with Z-VAD-FMK (Figure 2, A and B; and Supplemental Figures 2–4). To confirm that this was a necroptotic death, the RIP1 inhibitor necrostatin-1s was used. Importantly, reduction in cell viability and clonogenicity was abrogated by necrostatin-1s, indicating that the observed mode of cell death was necroptosis, not apoptosis (Figure 2, A and B; and Supplemental Figures 2–4). Further indication that this was not an apoptotic death is the inclusion of Z-VAD-FMK treatment, a pan-caspase inhibitor that blocks apoptosis, which enhanced cell death rather than prevented it, consistent with necroptosis.

Although studies by other groups indicate that the cytotoxic effects of SMAC mimetics are potentiated by death ligands such as TNFA and TRAIL in HNSCC (25, 27), we did not observe dramatic increases in birinapant-induced necroptosis in the presence of exogenous death ligands in the MOC1 or UMSCC 17A cell lines (Supplemental Figures 2 and 3), a phenomenon that might be explained by low activity of death receptor signaling in these lines (35).

Enhanced necroptotic cell death observed under knockdown of CASP8 was further evaluated with annexin V staining (36). Knockdown of CASP8 led to a significant increase in annexin V–positive staining following treatment of UMSCC 17A and MOC1 cells with birinapant alone and birinapant in combination with Z-VAD-FMK. Necrostatin-1s reversed positive annexin V staining induced by birinapant in combination with Z-VAD-FMK in both the cell lines, suggesting the predominant occurrence of RIP1-mediated, necroptotic cell death (Figure 2C and Supplemental Figure 5).

The expression of key cell death pathway markers following birinapant alone and Z-VAD-FMK combination treatments in the absence or presence of necrostatin-1s was evaluated by Western blotting (Figure 2D). Knockdown of CASP8 was associated with a significant increase in the protein levels of phospho-MLKL and phospho-RIP1 following birinapant alone and Z-VAD-FMK combination treatments in the UMSCC 17A and MOC1 cells, respectively. The increased phosphorylation was accompanied by a significant reduction in the total protein levels of RIP1, RIP3, and MLKL due to their proteosomal degradation at this 24-hour time point (37). Necrostatin-1s reversed the effects of birinapant alone and the birinapant and Z-VAD-FMK combination. Taken together, these data clearly demonstrate that loss of CASP8 sensitizes HNSCCs to birinapant and birinapant plus Z-VAD-FMK induced necroptotic cell death in vitro.

### Knockdown of CASP8 enhances the radiosensitizing effects of birinapant and Z-VAD-FMK through induction of necroptosis.

**Figure 3:**
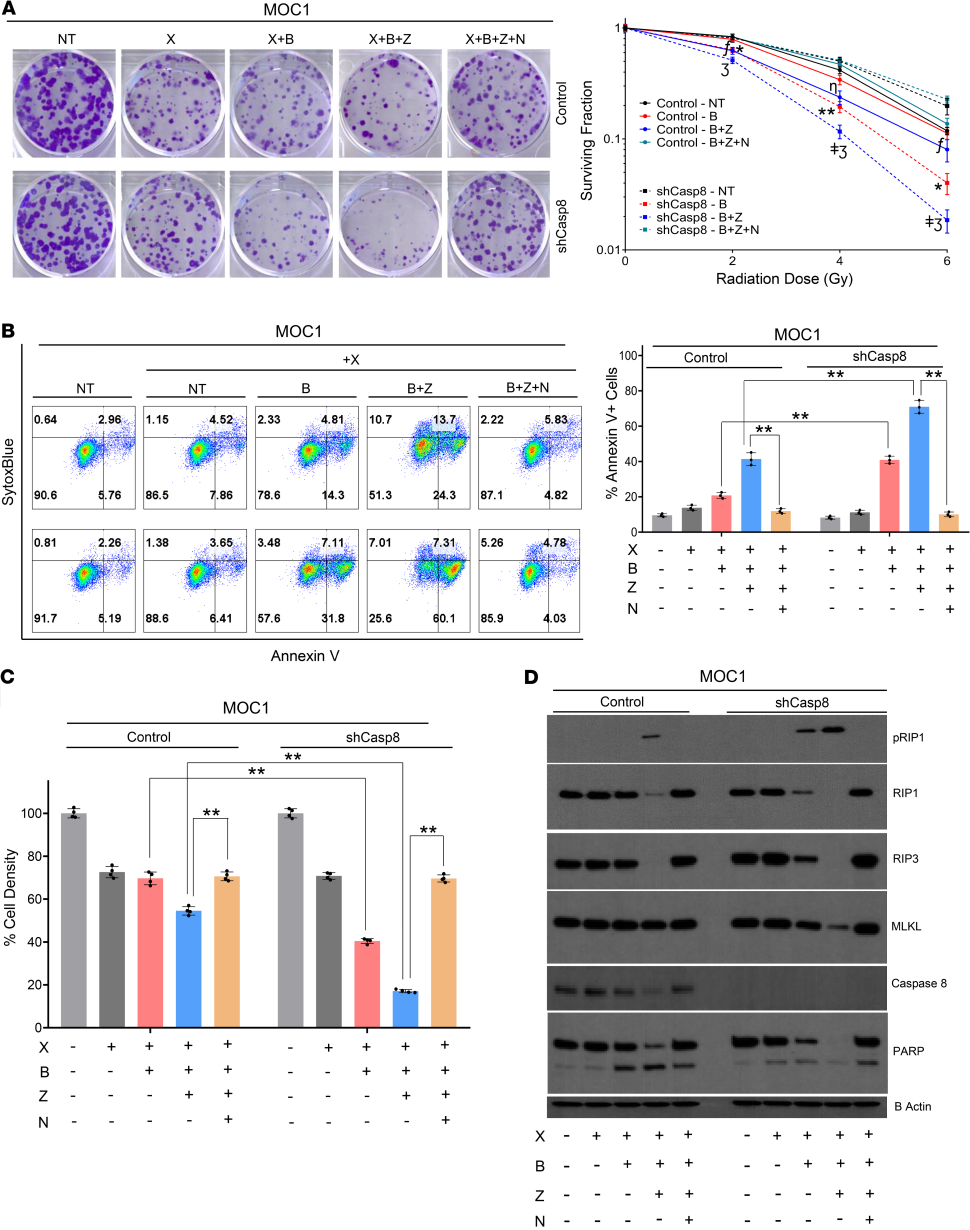
Knockdown of CASP8 enhances the radiosensitizing effects of birinapant and Z-VAD-FMK through induction of necroptosis. MOC1 control and shCasp8 cells were treated with radiation (X [2, 4, and 6 Gy]), birinapant (B [125 nmol/L in **A**; 250 nmol/L in **B**–**D**]), Z-VAD-FMK (Z [5 μmol/L]), necrostatin-1s (N [10 μmol/L]), or the combinations as indicated. (**A**) Representative images of clonogenic survival assays for the X (4 Gy) conditions. Twenty-four hours after treatments, drug dilutions were washed out, and colonies were allowed to form for 5 days, after which they were stained and counted. Surviving colony counts were normalized to nontreated cells (cells treated with no drugs) of each radiation dose from the same experiment. Log_10_ of surviving fractions were plotted. All treatments were carried out in triplicates. One-way ANOVA with post hoc Bonferroni-corrected *t* test was used for statistics. *P < 0.05 and **P < 0.001, when comparing surviving fractions following X+B treatments between control and shCASP8 cells. ^ǂ^*P* < 0.05, when comparing surviving fractions following X+B+Z treatments between control and shCasp8 cells. ^ƒ^*P* < 0.05, when showing reversal of death upon addition of N to X+B+Z for the control cells. ^ƞ^*P* < 0.001, when showing reversal of death upon addition of N to X+B+Z for the control cells. ^Ʒ^*P* < 0.001, when showing reversal of death upon addition of N to X+B+Z for the shCasp8 cells. Symbols are placed at the radiation doses they refer to. Please refer to Supplemental Figure 6 and Supplemental Table 6 for more detailed analysis. (**B**) Annexin V-APC/SytoxBlue staining was performed 24 hours after treatments. Cells were treated with X (6 Gy). Percentage of annexin V positivity was used as a measure to assess cell death. All treatments were carried out in triplicates. (**C**) Cell viability was assessed using CellTiter-Glo. Values normalized to nontreated cells from the same experiment to calculate percentage of cell density. All treatments were carried out in replicates of 4. X (6 Gy) data are shown for simplicity. A more detailed version can be found in Supplemental Figure 7; 1-way ANOVA with post hoc Bonferroni-corrected *t* test was used for statistics. *P < 0.05, **P < 0.001 for the indicated pairwise comparisons for **B** and **C**. (**D**) Whole-cell lysates were collected 24 hours after treatments (with X at 6 Gy) and subjected to Western blot analysis for the indicated key cell death markers; β-actin was used as loading control. All experiments detailed above were repeated 3 times with similar results.

The combination of SMAC mimetic birinapant and radiation has been shown to be active in HNSCCs with alterations in cell death pathways (25). Given *CASP8*-mutant HNSCC cell lines are more radioresistant than their WT counterparts and *CASP8*-mutant HNSCCs show alterations in the necroptosis pathway, we sought to understand whether necroptosis could be exploited therapeutically to treat HNSCCs with compromised *CASP8* status. To test this, shCasp8 and scrambled shRNA control MOC1 cells were treated with increasing doses of radiation in combination with birinapant and/or Z-VAD-FMK in the absence or presence of necrostatin-1s and assessed for clonogenic survival. Knockdown of Casp8 alone did not significantly influence radiosensitivity (Figure 3A and Supplemental Figure 6). The magnitude of the radiosensitizing effects of birinapant and birinapant plus Z-VAD-FMK was quantified by comparing the mean radiation doses required to reach a surviving fraction of 0.25 (SF_0.25_) for the control versus each of the drug treatment conditions and by calculating dose enhancement ratios (DERs), normalizing mean SF_0.25_ values for each drug treatment condition to its corresponding control (38). Interestingly, the radiosensitizing effects of birinapant (with a DER of 1.07 for the control vs. 1.53 for the shCasp8 cells) and birinapant plus Z-VAD-FMK (with a DER of 1.24 for the control and 1.81 for the shCasp8 cells) were enhanced under knockdown of Casp8. The addition of necrostatin-1s returned all colony counts to comparable levels to those treated with radiation alone, indicating that necroptosis is the underlying mechanism through which the cells were sensitized (Figure 3A, Supplemental Figure 6, and Supplemental Table 6). These results were further confirmed with assays for annexin V (Figure 3B) and cell viability (Figure 3C and Supplemental Figure 7) that demonstrated similar necroptotic radiosensitization. To further elaborate on the mechanism(s) that lead to cell death, we assayed the levels of key cell death proteins by Western blot following induction of necroptosis and radiation treatment (Figure 3D). Birinapant when combined with radiation led to phosphorylation of RIP1 in shCasp8 but not scrambled shRNA control MOC1 cells, a phenomenon that was accompanied by reduction in the protein levels of RIP1, RIP3, and MLKL, indicating activation of the necroptosis pathway. This phenotype was further enhanced by the addition of Z-VAD-FMK to the treatment and reversed by necrostatin-1s treatment, indicating that the radiosensitizing effects of birinapant and birinapant plus Z-VAD-FMK manifest themselves through induction of necroptosis.

### Susceptibility to necroptosis is determined by levels of RIP3 in HNSCCs.

**Figure 4:**
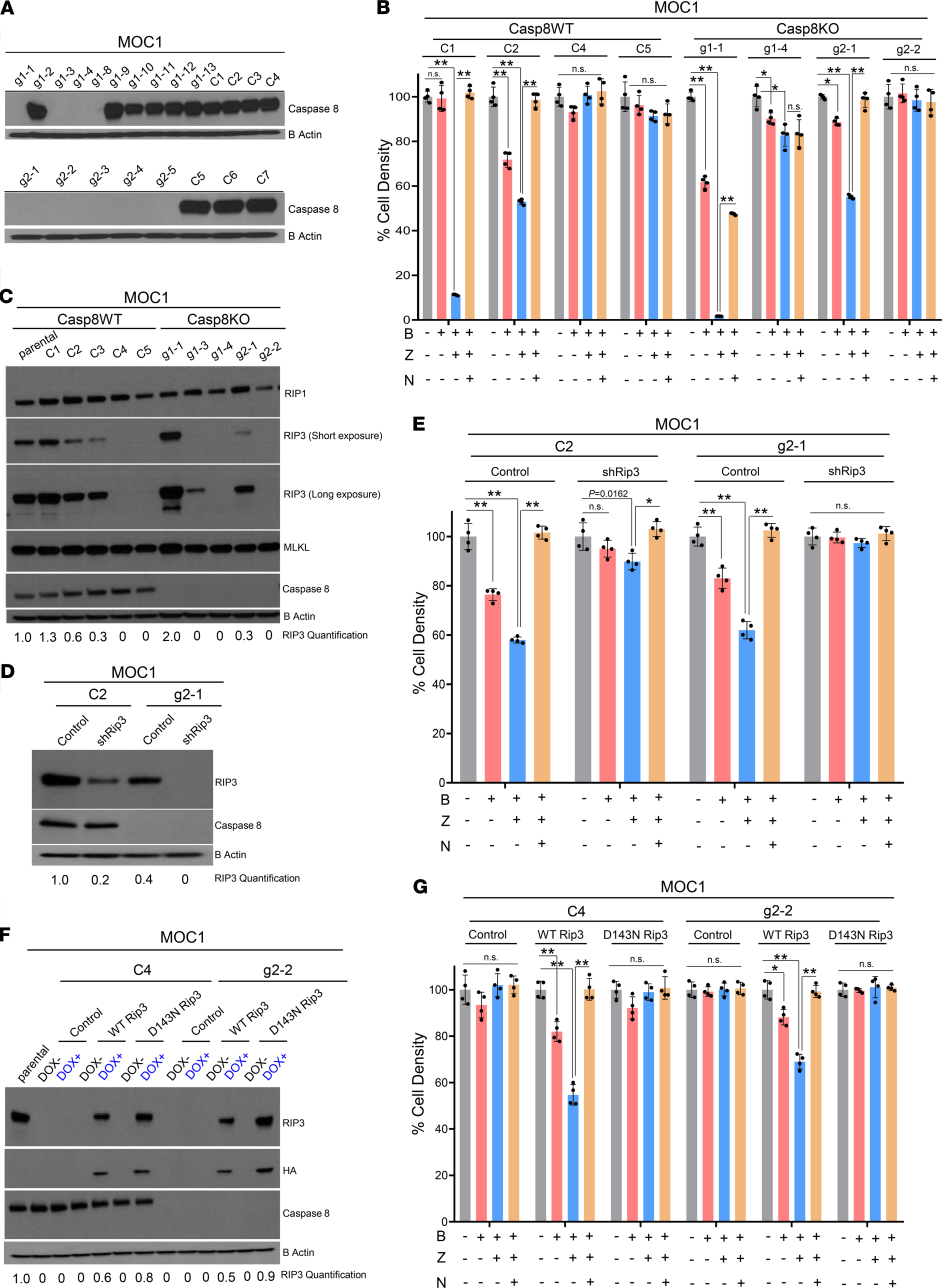
Susceptibility to necroptosis is determined by levels of RIP3 in HNSCCs. (**A**) CRISPR/Cas9 was used to knock out CASP8 in the mouse-derived MOC1 cell line. MOC1 parental cells were transiently transfected with 2 sgRNAs designed against mouse *Casp8* (sgRNA-mCASP8 #1 and sgRNA-mCASP8 #2) or a nontargeting sgRNA, after which clonal selection/expansion was performed. Engineered clones were subjected to a Western blot screen to identify Casp8WT and Casp8KO MOC1 clones. (**B**) Indicated Casp8WT and Casp8KO MOC1 clones were treated with birinapant (B [1 μmol/L]), Z-VAD-FMK (Z [5 μmol/L]), necrostatin-1s (N [10 μmol/L]), or the combinations for 24 hours. Cell viability was assessed using CellTiter-Glo. Values normalized to nontreated cells from the same experiment to calculate percentage of cell density. All treatments were carried out in replicates of 4. (**C**) Indicated Casp8WT and Casp8KO MOC1 clones were subjected to Western blot analysis for necroptosis markers RIP1, RIP3, and MLKL. (**D**) RIP3 was knocked down using shRNA in 2 necroptosis-sensitive MOC1 clones: the Casp8WT C2 and Casp8KO g2-1 clones. Scrambled shRNA control and shRip3 cells were subjected to Western blot to validate knockdown of RIP3. (**E**) Control and shRip3 C2 (Casp8WT) and g2-1 (Casp8KO) MOC1 clones were treated with birinapant (B [1 μmol/L]), Z-VAD-FMK (Z [5 μmol/L]), necrostatin-1s (N [10 μmol/L]), or the combinations for 24 hours. Cell viability was assessed by CellTiter-Glo. All treatments were carried out in replicates of 4. (**F**) Necroptosis-resistant C4 (Casp8WT) and g2-2 (Casp8KO) MOC1 clones were transduced with control, HA-tagged WT Rip3, or HA-tagged D143N Rip3 (a kinase domain dead RIP3) inducible expression constructs. RIP3 expression was induced with doxycycline (50 ng/mL). Western blot analysis was performed to validate expression of WT or D143N Rip3 in the indicated MOC1 clones. Relative RIP3 expression was quantified using parental cells in **C** and **F** and C2 control cells in **D** as reference control, and β-actin was used as loading control. (**G**) Cells engineered in **F** were treated with birinapant (B [1 μmol/L]), Z-VAD-FMK (Z [5 μmol/L]), necrostatin-1s (N [10 μmol/L]), or the combinations for 24 hours. Cell viability was assessed by CellTiter-Glo. One-way ANOVA with post hoc Bonferroni-corrected *t* test was used for statistics. *P < 0.05; **P < 0.001 for the indicated pairwise comparisons. All experiments detailed above were repeated 3 times with similar results.

The observation that Z-VAD-FMK enhances birinapant-induced necroptotic killing under knockdown of CASP8 might indicate inhibition of residual CASP8 activity. To further study how loss of CASP8 affects necroptosis sensitivity, we genetically deleted *Casp8* in MOC1 cells using CRISPR/Cas9. To rule out clone-specific or off-target effects, we designed 2 different single-guide RNAs (sgRNAs) against *Casp8* to generate multiple independent Casp8-knockout MOC1 clones. Matching Casp8 WT clones were created using a nontargeting sgRNA. The MOC1 CRISPR clones were subjected to Western blotting to confirm efficient knockout of Casp8 along with the parental cell line (Figure 4A). Necroptosis sensitivity was tested in 4 independent Casp8 WT and Casp8-knockout clones following treatment with birinapant or birinapant in combination with Z-VAD-FMK in the absence or presence of necrostatin-1s (Figure 4B). Across the clones tested, 2 Casp8 WT clones (C1 and C2) and 2 Casp8-knockout clones (g1-1 and g2-1) showed sensitivity to birinapant plus Z-VAD-FMK, with necrostatin-1s restoring cell density, indicating a predominantly RIP1-mediated necroptotic cell death. Intriguingly, however, among the Casp8-knockout clones, clone g2-2 showed complete resistance to birinapant and birinapant plus Z-VAD-FMK, and clone g1-4 demonstrated low sensitivity, which was not reversed by necrostatin-1s. These 2 lines exhibited necroptosis resistance despite complete loss of Casp8. Additionally, 2 control clones (C4 and C5) also demonstrated resistance to necroptosis. Western blot analysis of the whole-cell lysates obtained from the MOC1 clones and the parental cell line at baseline revealed considerable levels of RIP1 and MLKL for all the cells, but the clones with reduced sensitivity to necroptosis demonstrated lack of protein expression of RIP3 irrespective of Casp8 status, suggesting that RIP3 levels might determine necroptosis sensitivity in HNSCCs (Figure 4C).

We next conducted knockdown and overexpression studies to further validate the role of RIP3 in determining necroptosis sensitivity in HNSCCs. shRNA knockdown of Rip3 in 2 necroptosis-sensitive MOC1 clones, namely a Casp8 WT C2 and a Casp8-knockout g2-1 clone, resulted in acquisition of resistance to birinapant plus Z-VAD-FMK induced necroptotic cell death (Figure 4, D and E). Conversely, inducible expression of WT but not a kinase-dead (D143N) mutant of Rip3 in 2 necroptosis-resistant MOC1 clones, namely a Casp8 WT C4 and a Casp8-knockout g2-2 clone, rendered the cells sensitive to birinapant plus Z-VAD-FMK induced necroptotic cell death (Figure 4, F and G). Taken together, these data suggest that the presence of functional RIP3 is necessary for susceptibility to necroptosis in HNSCCs.

### RIP3 expression is silenced in many HNSCC cell lines, but patient tumors show considerable expression.

**Figure 5:**
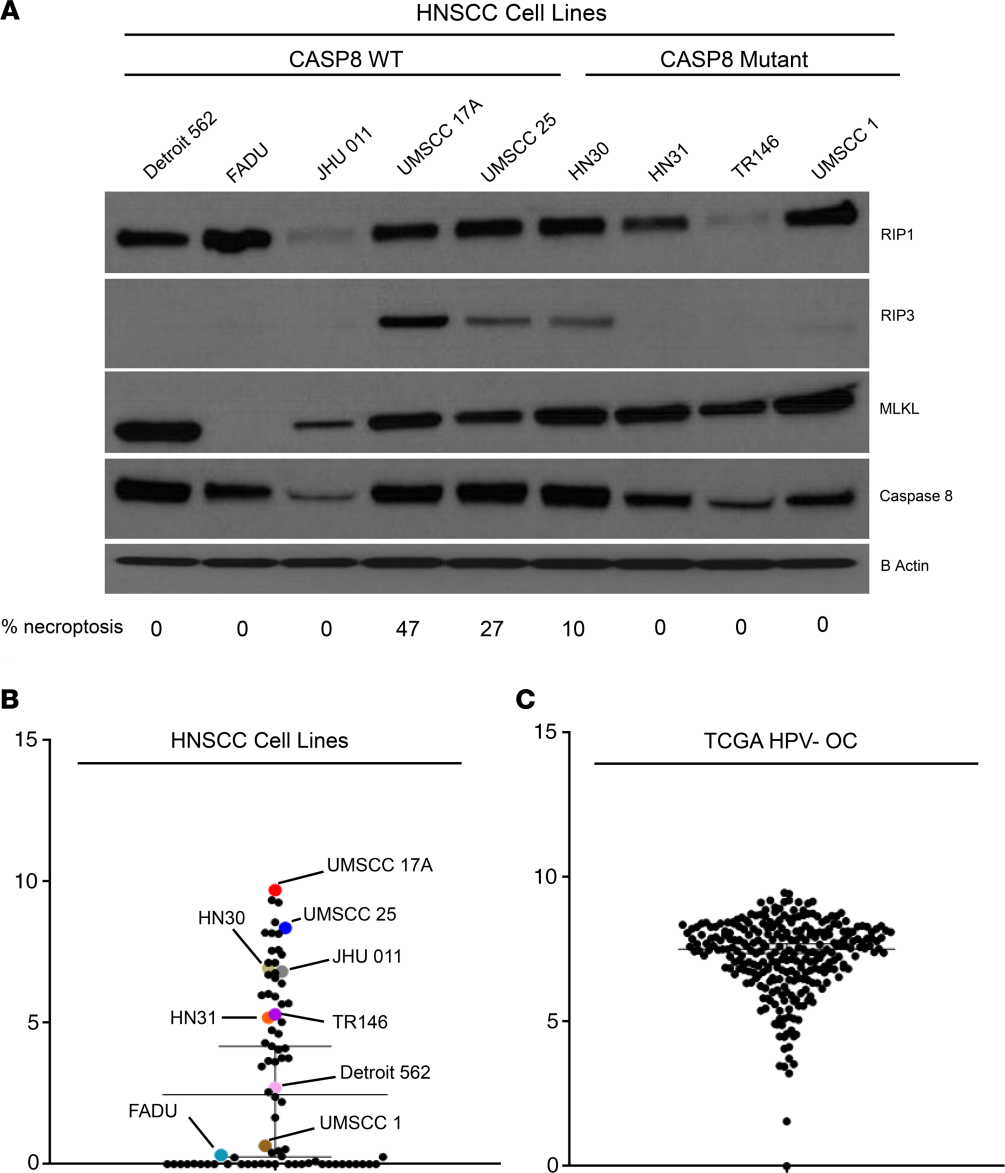
RIP3 expression is silenced in many HNSCC cell lines, but patient tumors show considerable expression. (**A**) Cell lysates obtained from a panel of 5 *CASP8*WT and 4 *CASP8*mutant human-derived HNSCC cell lines were subjected to Western blot analysis for caspase-8 and key necroptosis markers; β-actin was used as loading control. Cell lines were treated with birinapant (B [1 μmol/L]), Z-VAD-FMK (Z [5 μmol/L]), necrostatin-1s (N [10 μmol/L]), or the combinations for 24 hours. Cell viability was assessed using CellTiter-Glo. Then, “% necroptosis” was used to assess necroptosis sensitivity for each cell line and calculated based on percentage reduction in cell density with birinapant plus Z-VAD-FMK treatment that was reversible by necrostatin-1s. This is a measure of necroptotic death. Please refer to Supplemental Figure 8 for detailed analysis. (**B**) Scatter plot shows gene expression for RIP3 in a panel of 80 human-derived HNSCC cell lines. Median with 95% CI values are shown by the bar. Cell lines used for the Western blot analysis were highlighted. (**C**) RIP3 gene expression in TCGA HPV-negative oral cancer samples. Median with 95% CI values are shown by the bar.

The observation that MOC1 clones lacking functional Rip3 protein show necroptosis resistance led us to test whether RIP3 levels determine necroptosis sensitivity in other HNSCC cell lines. We took a panel of 5 *CASP8* WT and 4 *CASP8*-mutant HNSCC cell lines, tested their sensitivity to necroptosis induction, and determined the baseline expression of key necroptosis pathway proteins by Western blotting (Figure 5A and Supplemental Figure 9). Consistent with our previous findings, across all the cell lines tested, only 3 cell lines that demonstrated considerable baseline protein levels of RIP3, the *CASP8* WT UMSCC 17A and UMSCC 25 cell lines and the *CASP8*-mutant HN30 cell line, showed sensitivity to birinapant plus Z-VAD-FMK, which was reversible by necrostatin-1s, indicating induction of necroptotic cell death. We next evaluated RIP3 gene expression levels in HNSCC cell lines by using RNA sequencing (Figure 5B). Cell lines with detectable protein expression of RIP3 showed the highest RNA levels of *RIP3*, where many cell lines demonstrated low RIP3 expression, suggesting that low levels of RIP3 might underlie necroptotic resistance in HNSCC. Given our interest in the use of necroptosis as a therapeutic target in HNSCCs, we assessed RIP3 gene expression in HNSCC tumors using the publicly available TCGA HNSCC data set (Figure 5C). Analysis of TCGA tumors confirmed high levels of RIP3 in most patient samples, providing justification for therapeutic use of necroptosis in HNSCC. We hypothesize that the loss of RIP3 expression in many cell lines may be due to promoter DNA methylation that occurs in vitro (39, 40).

### Loss of CASP8 increases sensitivity to single-agent birinapant and birinapant plus radiation in vivo.

**Figure 6:**
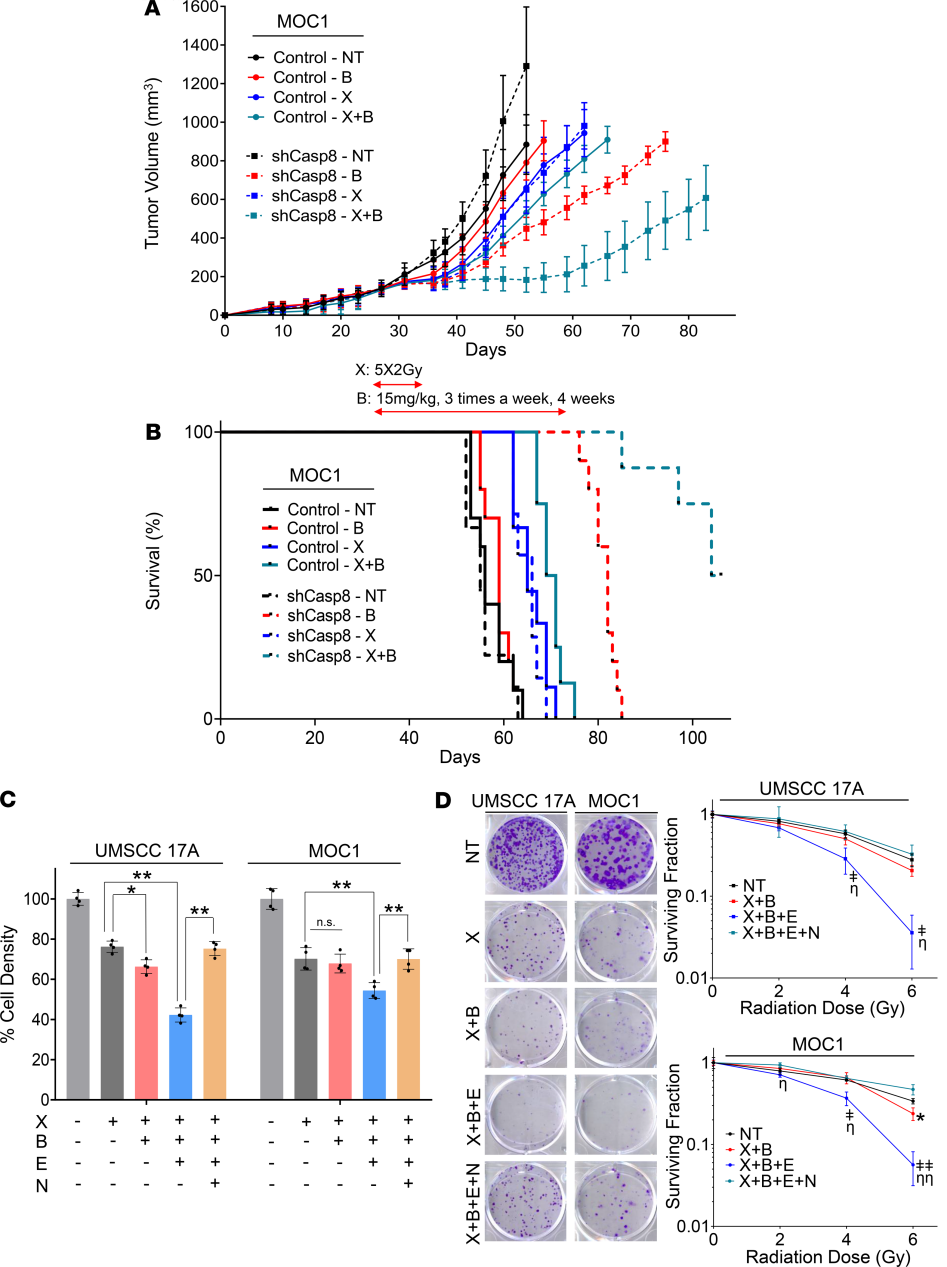
Loss of CASP8 increases sensitivity to single-agent birinapant and birinapant plus radiation in vivo. (**A**) 2 × 10^6^ MOC1 cells transduced with an inducible shRNA against *Casp8* were injected into the right flank of WT female C57BL/6 mice. Mice were randomized and placed on control or DOX diet 3 days after injection to induce knockdown of Casp8 in vivo (Supplemental Figure 9, WB images). Control and shCasp8 mice were randomized into 4 treatment groups (vehicle control [black lines], 15 mg/kg birinapant [red lines], 5 × 2 Gy radiation [blue lines] or combination [cerulean lines], *n* = 7–10/each) 27 days after inoculation when the average tumor volume reached ~150 mm^3^. Solid and dashed lines represent control and shCasp8 mice, respectively, for the indicated treatment groups (Supplemental Figure 10, schema). Error bars represent standard deviation. Two-way ANOVA was used for statistical analysis. *P < 0.05, **P < 0.001 for indicated pairwise comparisons (Table 1 and Supplemental Tables 7–9). (**B**) Kaplan-Meier survival curves representing each treatment group. Log-rank (Mantel-Cox) test was used for statistical analysis. *P < 0.05, **P < 0.001 for indicated pairwise comparisons. (**C**) UMSCC 17A and MOC1 parental cells were treated with radiation (X [2, 4, and 6 Gy]), birinapant (B [50 nmol/L for the UMSCC-17A cells; 250 nmol/L for the MOC1 cells]), emricasan (E [1 μmol/L for both the cell lines]), necrostatin-1s (N [10 μmol/L for both the cell lines]), or the combinations as indicated for 24 hours. Cell viability was assessed using CellTiter-Glo as per Methods. One-way ANOVA with post hoc Bonferroni-corrected *t* test was used for statistics. *P < 0.05, **P < 0.001 for indicated pairwise comparisons. X (6 Gy) data are shown for simplicity (Supplemental Figure 11). (**D**) Representative images of clonogenic survival assays for the X (6 Gy) conditions. Clonogenic survival assay was performed as detailed in the Methods (birinapant doses reduced to 25 nmol/L and 125 nmol/L for the UMSCC 17A and MOC1 cells, respectively, for this assay, and other drugs used at the same concentrations as stated above). One-way ANOVA with post hoc Bonferroni-corrected *t* test was used for statistics. *P < 0.05, when comparing X+B to X alone for indicated radiation dose. ^ǂ^*P* < 0.05 and ^ǂǂ^*P* < 0.001, when comparing X+B+E to X alone for the indicated radiation doses. ^ƞ^*P* < 0.05 and ^ƞƞ^*P* < 0.001, when comparing X+B+E to X+B+E+N for the indicated radiation doses (Supplemental Figure 12). Experiments detailed above were repeated 3 times with similar results.

On the basis of our in vitro observations that loss of Casp8 increased birinapant sensitivity and enhanced the radiosensitizing effects of birinapant in MOC1 cells, we sought to assess the therapeutic efficacy of birinapant alone or in combination with radiation in the absence or presence of Casp8 knockdown using the syngeneic MOC1 model in vivo. To test this, we generated a MOC1 cell subline transduced with a doxycycline-inducible lentiviral Casp8 shRNA vector (41). Treatment of the Casp8 shRNA-MOC1 cells with doxycycline, but not vehicle control, led to efficient knockdown of Casp8 in vitro (Supplemental Figure 9A). The Casp8 shRNA-MOC1 cells were injected subcutaneously into the upper leg of syngeneic C57BL/6 mice, and knockdown of Casp8 was achieved in vivo by feeding the animals doxycycline-containing food, or matching control diet for control animals, throughout the study (Supplemental Figure 9B). Mouse cohorts with control and Casp8-knockdown MOC1 flank tumors were treated with birinapant (15 mg/kg intraperitoneally) every 3 days for 4 weeks, radiation 2 Gy daily Monday to Friday for 1 week, or the combination when the tumors reached 150 mm^3^ (25). The schema of in vivo treatments is shown in Supplemental Figure 10. Treatment with radiation alone significantly inhibited in vivo tumor growth (Figure 6A, Table 1, and Supplemental Table 7) and improved survival (Figure 6B, Table 1, and Supplemental Table 8) in both the control and Casp8-knockdown animal cohorts. However, single-agent birinapant proved effective in delaying tumor growth only when combined with Casp8 knockdown, a phenomenon that was accompanied by a significant survival benefit. The radiation plus birinapant combination further reduced in vivo tumor growth and provided survival benefit in both the control and Casp8-knockdown animal cohorts. The degree of radiosensitization achieved with birinapant was assessed by calculating radiation enhancement factor (REF) for the control and shCasp8 mouse cohorts treated with radiation in combination with birinapant, taking into account the mean tumor doubling times from the day of randomization (day 27) for all the groups (42). (Please refer to Supplemental Table 9 for the calculations.) Interestingly, birinapant enhanced sensitivity to radiation only under Casp8 knockdown (with an REF of 0.5 for the control vs. 2.3 for the shCasp8 mouse cohorts), with mice bearing shCasp8 MOC1 tumors demonstrating a significant increase in tumor growth delay and a significantly improved survival when compared with those bearing matching control tumors (Figure 6, A and B; Table 1; and Supplemental Tables 7–9). Taken together, our results suggest that loss of CASP8 sensitizes HNSCCs to birinapant and potentiates its radiosensitizing effects in vivo.

### Chemical inhibition of CASP8 sensitizes to birinapant plus radiation.

Since only a subset of HNSCCs contain inactivation of *CASP8* by mutation, we sought to determine whether chemical inhibition of CASP8 could mimic knockdown of CASP8 for necroptotic radiosensitization. Emricasan (IDN 655) is a potent pan-caspase inhibitor that was well tolerated in patients when it was tested for reduction of liver toxicity in patients with liver diseases (43). We found that emricasan sensitized *CASP8* WT MOC1 and UMSCC 17A cells to necroptosis when combined with XRT and birinapant (Figure 6, C and D; and Supplemental Figures 11 and 12), suggesting that targeting the necroptosis pathway through chemical inhibition of CASP8 could be a therapeutic strategy to radiosensitize HNSCCs.

## Discussion

In this study, we found that *CASP8* status regulates necroptotic death in HNSCC, and SMAC mimetic treatment may be useful to exploit this pathway for therapeutic benefit. SMAC mimetics have shown therapeutic potential in a variety of cancers, including HNSCC, through induction of cancer cell death directly or via synergistic interaction with other cytotoxic therapies, such as chemotherapy, radiotherapy, or immunotherapies (18–24). Previous studies of HNSCC have reported that SMAC mimetics, including birinapant, might synergize with radiation to delay tumor growth in various xenograft models (25–27). We show for the first time to our knowledge that inhibition of CASP8 function can lead to enhanced radiosensitization by birinapant through induction of necroptotic death (Figure 3, A–D; Supplemental Figures 6 and 7; and Supplemental Table 6). Since *CASP8*-mutant HNSCCs might be more radioresistant than their WT counterparts (Figure 1B), combining SMAC mimetics with radiation to induce necroptosis is a potentially promising therapeutic strategy to improve radiation response in HNSCCs with compromised *CASP8* status. Since *CASP8* mutations in HNSCC are often heterozygous (4), further study is necessary to determine whether dominant-negative or gain-of-function phenotypes may be present.

Although previous reports have demonstrated that SMAC mimetics alone or in combination with radiation can suppress tumor cell growth in *CASP8* WT HNSCC through induction of apoptosis (25, 27), we did not identify a large apoptotic component in most of the cell lines we analyzed. Rather, the apoptotic inhibitor Z-VAD-FMK enhanced cell death in our studies. This discrepancy may be due to the use of different HNSCC cell lines that reflect the genomic diversity of HNSCC. However, it may also indicate the broad therapeutic potential of these treatment combinations. It is likely that many HNSCCs may be sensitive to some type of cell death induced by a SMAC mimetic alone or in combination with radiation. The *CASP8* status and other genomic alterations (*FADD/BIRC2/BIRC3* amplification, RIP3 expression) may determine whether the death is necroptotic or apoptotic, but many genotypes will be sensitive. Additionally, we found that the mode of cell death for *CASP8* WT cells can be pushed toward necroptosis by adding treatment with a caspase inhibitor, such as emricasan. The ability to tailor the mode of cell death could facilitate therapeutic synergy with other treatment agents, including immunotherapy.

Immunotherapy is an exciting new treatment modality for HNSCC and many other tumor types; however, only a minority of patients respond. Some tumors seems to have an immunologically cold microenvironment that prevents an immunologic response (44). It has been argued, and recently demonstrated (45), that activation of necroptotic death can potentiate antitumor immunity. Necroptosis is a more immunogenic form of cell death, which could be detrimental to healthy tissues but may be useful as a treatment for cancer. Therefore, it is attractive to speculate that the treatments we have investigated could enhance responses to immunotherapy by inducing an immunogenic necroptotic death.

Caspase inhibition has generally not been thought of as a useful therapeutic approach for cancer because the goal is to promote cell death rather than block it. However, a previous report showed that the caspase inhibitor emricasan renders clinically relevant models of acute myeloid leukemia susceptible to birinapant-induced necroptosis in vitro and in vivo (46). In our study, emricasan significantly enhanced the radiosensitizing effects of birinapant in 2 preclinical models of HNSCC, through induction of necroptosis (Figure 6, C and D; and Supplemental Figures 11 and 12). Emricasan is well tolerated in patients and is being tested in humans for the treatment of liver diseases characterized by hepatic inflammation and fibrosis (47). It is interesting to propose the combination of birinapant (or other SMAC mimetics such as ASTX660 or LCL161) and radiation with emricasan to inhibit apoptotic death but promote necroptotic death, thereby using caspase inhibition for cancer therapy.

Presence of RIP3 has been shown to be pivotal in determining the sensitivity of a variety of cancer types to necroptosis (34). In that study, the cancer cell lines that showed lack of RIP3 expression were found to be resistant to the combination of TNFA, Z-VAD-FMK, and the SMAC mimetic SM164. Similarly, in another study where 8 colon cancer cell lines were tested for sensitivity to a TNFA, SMAC mimetic, and Z-VAD-FMK combination, only those that were devoid of RIP3 at the mRNA and protein levels failed to undergo necroptosis (48). Consistent with these results, our data suggest that *RIP3* loss can be an underlying mechanism by which HNSCCs become resistant to necroptotic death stimulated with birinapant and Z-VAD-FMK (Figure 4, A–G). Loss of protein expression of RIP3 in necroptosis-resistant HNSCCs coincided with low mRNA levels of *RIP3* (Figures 5, A and B; Supplemental Figure 8), indicating a likely transcriptional regulation of RIP3. Intriguingly, in a study where mechanisms of RIP3 loss were investigated in various cancer cell lines, treatment of RIP3-lacking cells with the hypomethylating agent 5-aza-2′-deoxycytidine but not the proteosome inhibitor MG132 restored RIP3 expression. Further analyses conducted by the authors revealed that RIP3 loss in those cells was associated with methylation of 4 CpG islands located downstream of the transcription start site of *Rip3* (39). Therefore, it is likely that DNA methylation is the underlying mechanism for loss of RIP3 in the preclinical models of HNSCC that we employed in our studies. Since cell lines grown in 2D culture are prone to increased DNA methylation (40), we evaluated *RIP3* gene expression in HNSCC patient samples using TCGA HNSCC data set (Figure 5C). Analysis of TCGA tumors revealed that patient samples showed high levels of *RIP3*. Although this expression could potentially be from stromal or immune cells in the tumors, we did not find any correlations with other markers of stromal or immune cells, suggesting it originated in the tumor cells. The high levels of *RIP3* observed in patient tumors might provide justification for exploitation of necroptosis therapeutically in HNSCC, and could indicate that the silencing of RIP3 in some cell lines may be an artifact of 2D culture.

In conclusion, here we demonstrate that inhibition of CASP8 function enhances sensitivity of HNSCCs to birinapant and birinapant plus radiation through induction of necroptosis in vitro and in vivo, on the condition that RIP3 function is maintained. These results provide a strong clinical relevance for the combination of SMAC mimetics like birinapant and radiation in *CASP8*-mutant HNSCCs, a therapeutic approach that might potentially be effective even in *CASP8* WT patients with the use of a clinically tolerable caspase inhibitor, such as emricasan. Further studies to identify optimal and effective combination dose of emricasan with birinapant and/or radiation in vivo are warranted as are combinations with immunotherapy.

## Methods

### HNSCC cell lines.

Human-derived HNSCC cell lines were maintained as previously described (49). MOC1 cell line was provided by R. Uppaluri (Washington University School of Medicine, St. Louis, Missouri, USA) (33). All cell lines were authenticated by short tandem repeat profiling and cultured for no longer than 15 passages before use in experiments (49). Please refer to Supplemental Table 2 for cell line details.

### Genomic analysis.

TCGA data were obtained from TCGA Pan-CancerAtlas (50) and analyzed for *CASP8* mutations and RIP3 expression in HNSCC. Cell line *RIP3* gene expression data are available through the National Center for Biotechnology Information’s Gene Expression Omnibus (accession GSE122512) (51).

### Engineering of stable cell lines.

Cell lines were transduced with lentiviral shRNA constructs against *CASP8* (shCASP8) or control scrambled shRNA, containing GFP and puromycin resistance gene (GE Dharmacon). shCASP8 and control cells were GFP-sorted and subjected to puromycin selection (1 μg/mL). After antibiotic treatment, control and shCASP8 cells were assessed for protein expression of CASP8 by Western blotting (WB). CRISPR/Cas9 sgRNA constructs were designed to knock out Casp8 in MOC1 cells (52). Knockout of Casp8 was validated via WB screening and sequencing.

Rip3 was knocked down using lentiviral shRNA constructs (GE Dharmacon) in select MOC1 clonal cell lines carrying WT RIP3, as described above for CASP8. Select MOC1 clones that showed lack of protein expression of Rip3 were transduced with inducible lentiviral vectors encoding WT (plasmid 73701) or kinase-dead mutant (D143N) Rip3 (plasmid 73703) purchased from Addgene (these plasmids were gifted by F. Chen, University of Massachusetts Medical School, Worcester, Massachusetts, USA). Control pTRIPZ empty vector was obtained from MD Anderson Functional Genomics Core Facility. MOC1 cells transduced with control or Rip3 expression constructs were subjected to puromycin selection (1 μg/mL). Expression of WT or D143N Rip3 was induced by doxycycline (1 μg/mL) for 72 hours and verified by WB.

For the in vivo experiments, inducible lentiviral shRNA constructs were designed against luciferase (shLUC) or *Casp8* (shCasp8) in collaboration with the MD Anderson Institute for Applied Cancer Science as described previously (41). MOC1 cells transduced with shLUC or shCasp8 vectors were subjected to puromycin selection (1 μg/mL), treated with doxycycline (50 ng/mL) for 72 hours, and verified by WB. shRNA/sgRNA oligonucleotide sequences are available in Supplemental Table 3.

### Cell proliferation and viability assays.

To evaluate cell proliferation, HNSCC cells were plated in 96-well plates at 2 densities: 50 cells/well and 100 cells/well. Cell density was measured using CellTiter-Glo (Promega). Luminescence reads were taken at the indicated time points and normalized to day 0 reads to calculate cell doublings.

To assess cell viability following drug treatments, 3 × 10^3^ to 10 × 10^3^ cells were plated in 96-well plates and allowed to attach overnight. Cells were then treated with 0.01% DMSO (mock treatment) or birinapant, Z-VAD-FMK (or Emricasan), TNFA (or TRAIL), and necrostatin-1s either alone or in combinations at the indicated doses for 24 hours, after which cell viability was measured by CellTiter-Glo. Average luminescence values taken for each treatment condition were normalized to those of mock-treated cells from the same experiment to calculate percentage of cell density. All treatments were carried out in triplicates or greater. All experiments were repeated 3 times with similar results. Please refer to Supplemental Table 4 for detailed information about the drugs/reagents used in the study.

### Clonogenic survival assays.

HNSCC cells were seeded in 6-well plates at predetermined densities and allowed to adhere overnight. The next day, cells were treated with 0.01% DMSO (mock treatment) or birinapant, Z-VAD-FMK (or emricasan), and necrostatin-1s either alone or in combinations at the indicated doses. For radiosensitivity assays, treatments described above were given 1 hour before exposure to 2, 4, or 6 Gy radiation. Twenty-four hours after treatments, media containing the drug dilutions were aspirated and replaced with fresh media. Colonies were allowed to form for 5–12 days, after which they were fixed in methanol and stained with crystal violet (2%). Wells containing surviving colonies were scanned, and colonies with more than 50 cells were counted with the guidance of ImageJ software (NIH). The number of surviving colonies per well was calculated for each treatment condition and normalized to that of mock-treated cells from the same experiment to calculate percentage of colony counts. For the radiosensitivity assays, average surviving colony counts were normalized to those of mock-treated cells of each radiation dose from the same experiment to calculate surviving fractions. Log_10_ of surviving fractions were plotted. All treatments were carried out in triplicates. All experiments were repeated 3 times with similar results.

### Annexin V assays.

Annexin V staining was performed in accordance with the manufacturer’s guidelines (BD Biosciences). Briefly, 2.5 × 10^5^ to 7.5 × 10^5^ cells were seeded in 6 cm dishes and allowed to adhere overnight. The next day, cells were treated as indicated. All treatments were carried out in triplicates. Twenty-four hours after treatments, medium was collected from the dishes and set aside to keep any floating cells. Adherent cells were harvested with trypsin and added to the previously collected medium. The cells were then centrifuged at room temperature for 3 minutes at 1000 rpm, washed once in cold PBS, and stained with annexin V APC (BD Biosciences) and 1 μmol/L SytoxBlue (Thermo Fisher Scientific) in annexin V binding buffer. Samples were mixed gently and incubated at room temperature for 30 minutes, after which they were subjected to flow cytometry analysis. Flow data were analyzed using FlowJo software. All treatments were carried out in triplicates. All experiments were repeated 3 times with similar results.

### Western blotting.

2.5 × 10^5^ to 7.5 × 10^5^ HNSCC cells were seeded in 6 cm dishes and allowed to adhere overnight. The next day, cells were treated as indicated. For radiosensitization studies, cells were exposed to 6 Gy radiation with drug treatments being given 1 hour before exposure of cells to radiation. Twenty-four hours after treatments, whole-cell lysates were obtained, and Western blot analysis was carried out as previously described (4). ImageJ software was used to quantify select Western blots. All experiments were repeated 3 times with similar results. Please refer to Supplemental Table 5 for the list of Western blot antibodies.

### In vivo xenograft model.

Two million MOC1 cells transduced with an inducible shRNA against *Casp8* were injected into the right flank of WT female C57BL/6 mice obtained from Envigo/Harlan Labs in the presence of Matrigel (Corning). Mice were randomized 3 days after inoculation and placed on control (Global 18% Protein Rodent Diet) or DOX diet (doxycycline hyclate added at 625 mg/kg) obtained from Envigo to induce knockdown of Casp8 in vivo. Control and shCasp8 mice were randomized into 4 treatment groups (*n* = 7–10 mice/each) 27 days after injection of cells, when the average tumor volume reached approximately 150 mm^3^. Treatments included birinapant (15 mg/kg birinapant, every 3 days for 4 weeks, intraperitoneally, given 1 hour before exposure of mice to radiation), radiation (5 × 2 Gy, Monday to Friday for 1 week), or the combination (a detailed in vivo treatment schema is available in Supplemental Figure 11). Tumor measurements and mouse body weights were collected 3 times a week. No significant weight loss (>5% of total body weight) was observed in mice that might be associated with birinapant or radiation toxicity. Animal welfare was monitored daily by the animal facility staff, and mice were euthanized when tumor burden reached more than 1.5 cm in any dimension. Tumor samples were collected from a subset of control and shCasp8 mice that were not recruited in the drug treatment study. These tumor samples were minced and cultured in medium for 48 hours. Cells shed from the tumors that had attached to culture dishes were collected, lysed, and subjected to WB analysis for Casp8.

### Statistics.

Kaplan-Meier method was used to calculate overall survival for the patient population. *P* values used to compare gene expression levels for necroptosis markers between different patient cohorts were computed using the Mann-Whitney *U* test. One-way ANOVA with post hoc Bonferroni-corrected *t* test and 2-tailed Student’s *t* tests were conducted to analyze in vitro data. For the in vivo studies, a 2-way ANOVA test was used to make tumor volume comparisons between animal cohorts. Differences in survival rates between groups were compared using the log-rank (Mantel-Cox) test. All data were presented as mean ± SD unless otherwise noted. *P* values less than 0.05 were considered statistically significant.

### Study approval.

All in vivo experiments were carried out with approval of the IACUC at MD Anderson.

## Author contributions

CRP conceived and supervised the study. CRP and BU designed the experiments and interpreted the data with input from LW, SJF, FOGN, HDS, AGS, and JNM. Experiments were carried out by BU, MG, AL, KE, LW, EL, MZ, and JN. CRP and BU wrote the manuscript. The manuscript was reviewed by all the authors. CRP supervised design of experiments, data analysis, and preparation of the manuscript. CRP accepts responsibility for the integrity of the data and the accuracy of the data analysis.

## Supplementary Material

supplemental data

## Figures and Tables

**Table 1:**
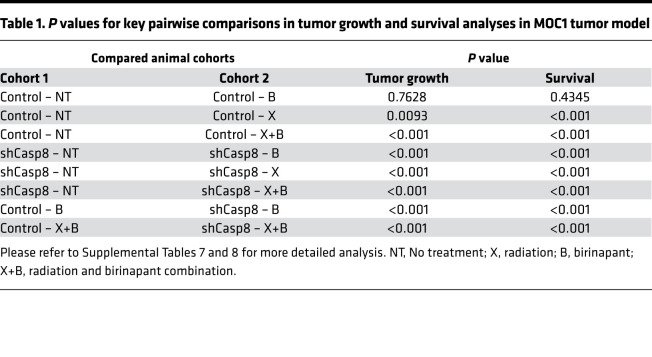
*P* values for key pairwise comparisons in tumor growth and survival analyses in MOC1 tumor model

## Version 1 10/27/2020

In-Press Preview

## Version 2 12/03/2020

Electronic publication

## References

[1] Bray F, Ferlay J, Soerjomataram I, Siegel RL, Torre LA, Jemal A (2018). Global cancer statistics 2018: GLOBOCAN estimates of incidence and mortality worldwide for 36 cancers in 185 countries. CA Cancer J Clin.

[2] Ang KK (2010). Human papillomavirus and survival of patients with oropharyngeal cancer. N Engl J Med.

[3] Agrawal N (2011). Exome sequencing of head and neck squamous cell carcinoma reveals inactivating mutations in NOTCH1. Science.

[4] Pickering CR (2013). Integrative genomic characterization of oral squamous cell carcinoma identifies frequent somatic drivers. Cancer Discov.

[5] Muzio M (1996). FLICE, a novel FADD-homologous ICE/CED-3-like protease, is recruited to the CD95 (Fas/APO-1) death--inducing signaling complex. Cell.

[6] Chinnaiyan AM, O’Rourke K, Tewari M, Dixit VM (1995). FADD, a novel death domain-containing protein, interacts with the death domain of Fas and initiates apoptosis. Cell.

[7] Fu TM (2016). Cryo-EM structure of caspase-8 tandem DED filament reveals assembly and regulation mechanisms of the death-inducing signaling complex. Mol Cell.

[8] Keller N, Mares J, Zerbe O, Grütter MG (2009). Structural and biochemical studies on procaspase-8: new insights on initiator caspase activation. Structure.

[9] Jäger R, Zwacka RM (2010). The enigmatic roles of caspases in tumor development. Cancers (Basel).

[10] Teitz T (2000). Caspase 8 is deleted or silenced preferentially in childhood neuroblastomas with amplification of MYCN. Nat Med.

[11] Pingoud-Meier C (2003). Loss of caspase-8 protein expression correlates with unfavorable survival outcome in childhood medulloblastoma. Clin Cancer Res.

[12] Kaiser WJ (2011). RIP3 mediates the embryonic lethality of caspase-8-deficient mice. Nature.

[13] Lin Y, Devin A, Rodriguez Y, Liu ZG (1999). Cleavage of the death domain kinase RIP by caspase-8 prompts TNF-induced apoptosis. Genes Dev.

[14] Feng S (2007). Cleavage of RIP3 inactivates its caspase-independent apoptosis pathway by removal of kinase domain. Cell Signal.

[15] Darding M (2011). Molecular determinants of Smac mimetic induced degradation of cIAP1 and cIAP2. Cell Death Differ.

[16] Dillon CP (2014). RIPK1 blocks early postnatal lethality mediated by caspase-8 and RIPK3. Cell.

[17] Tummers B, Green DR (2017). Caspase-8: regulating life and death. Immunol Rev.

[18] Fulda S (2015). Promises and challenges of Smac mimetics as cancer therapeutics. Clin Cancer Res.

[19] Petersen SL (2007). Autocrine TNFalpha signaling renders human cancer cells susceptible to Smac-mimetic-induced apoptosis. Cancer Cell.

[20] Probst BL (2010). Smac mimetics increase cancer cell response to chemotherapeutics in a TNF-α-dependent manner. Cell Death Differ.

[21] Dineen SP (2010). Smac mimetic increases chemotherapy response and improves survival in mice with pancreatic cancer. Cancer Res.

[22] Vellanki SH (2009). Small-molecule XIAP inhibitors enhance gamma-irradiation-induced apoptosis in glioblastoma. Neoplasia.

[23] Giagkousiklidis S, Vellanki SH, Debatin KM, Fulda S (2007). Sensitization of pancreatic carcinoma cells for gamma-irradiation-induced apoptosis by XIAP inhibition. Oncogene.

[24] Bake V, Roesler S, Eckhardt I, Belz K, Fulda S (2014). Synergistic interaction of Smac mimetic and IFNα to trigger apoptosis in acute myeloid leukemia cells. Cancer Lett.

[25] Eytan DF (2016). SMAC mimetic birinapant plus radiation eradicates human head and neck cancers with genomic amplifications of cell death genes FADD and BIRC2. Cancer Res.

[26] Yang L (2019). LCL161, a SMAC-mimetic, preferentially radiosensitizes human papillomavirus-negative head and neck squamous cell carcinoma. Mol Cancer Ther.

[27] Xiao R (2019). Dual antagonist of cIAP/XIAP ASTX660 sensitizes HPV^-^ and HPV^+^ head and neck cancers to TNFα, TRAIL, and radiation therapy. Clin Cancer Res.

[28] Kalu NN (2017). Genomic characterization of human papillomavirus-positive and -negative human squamous cell cancer cell lines. Oncotarget.

[29] Skinner HD (2012). TP53 disruptive mutations lead to head and neck cancer treatment failure through inhibition of radiation-induced senescence. Clin Cancer Res.

[30] Nehs MA (2011). Necroptosis is a novel mechanism of radiation-induced cell death in anaplastic thyroid and adrenocortical cancers. Surgery.

[31] Wang HH (2018). Ablative hypofractionated radiation therapy enhances non-small cell lung cancer cell killing via preferential stimulation of necroptosis in vitro and in vivo. Int J Radiat Oncol Biol Phys.

[32] Zhou W, Yuan J (2014). SnapShot: necroptosis. Cell.

[33] Judd NP (2012). ERK1/2 regulation of CD44 modulates oral cancer aggressiveness. Cancer Res.

[34] Najafov A (2018). BRAF and AXL oncogenes drive RIPK3 expression loss in cancer. PLoS Biol.

[35] Hannes S, Abhari BA, Fulda S (2016). Smac mimetic triggers necroptosis in pancreatic carcinoma cells when caspase activation is blocked. Cancer Lett.

[36] Lee EW (2012). Ubiquitination and degradation of the FADD adaptor protein regulate death receptor-mediated apoptosis and necroptosis. Nat Commun.

[37] Cai Z (2014). Plasma membrane translocation of trimerized MLKL protein is required for TNF-induced necroptosis. Nat Cell Biol.

[38] Raju U (2015). Inhibition of EGFR or IGF-1R signaling enhances radiation response in head and neck cancer models but concurrent inhibition has no added benefit. Cancer Med.

[39] Koo GB (2015). Methylation-dependent loss of RIP3 expression in cancer represses programmed necrosis in response to chemotherapeutics. Cell Res.

[40] Morgan MJ, Kim YS (2015). The serine threonine kinase RIP3: lost and found. BMB Rep.

[41] Sigl R, Ploner C, Shivalingaiah G, Kofler R, Geley S (2014). Development of a multipurpose GATEWAY-based lentiviral tetracycline-regulated conditional RNAi system (GLTR). PLoS One.

[42] Milas L (1999). Enhancement of tumor radioresponse in vivo by gemcitabine. Cancer Res.

[43] Pockros PJ (2007). Oral IDN-6556, an antiapoptotic caspase inhibitor, may lower aminotransferase activity in patients with chronic hepatitis C. Hepatology.

[44] Bonaventura P (2019). Cold tumors: a therapeutic challenge for immunotherapy. Front Immunol.

[45] Snyder AG (2019). Intratumoral activation of the necroptotic pathway components RIPK1 and RIPK3 potentiates antitumor immunity. Sci Immunol.

[46] Brumatti G (2016). The caspase-8 inhibitor emricasan combines with the SMAC mimetic birinapant to induce necroptosis and treat acute myeloid leukemia. Sci Transl Med.

[47] Mehta G (2018). A placebo-controlled, multicenter, double-blind, phase 2 randomized trial of the pan-caspase inhibitor emricasan in patients with acutely decompensated cirrhosis. J Clin Exp Hepatol.

[48] Yang C (2017). Regulation of RIP3 by the transcription factor Sp1 and the epigenetic regulator UHRF1 modulates cancer cell necroptosis. Cell Death Dis.

[49] Zhao M (2011). Assembly and initial characterization of a panel of 85 genomically validated cell lines from diverse head and neck tumor sites. Clin Cancer Res.

[50] Campbell JD (2018). Genomic, pathway network, and immunologic features distinguishing squamous carcinomas. Cell Rep.

[51] Gleber-Netto FO (2019). Variations in HPV function are associated with survival in squamous cell carcinoma. JCI Insight.

[52] Ran FA, Hsu PD, Wright J, Agarwala V, Scott DA, Zhang F (2013). Genome engineering using the CRISPR-Cas9 system. Nat Protoc.

