# Supplementary material for: Caspase-8 loss radiosensitizes head and neck squamous cell carcinoma to SMAC mimetic–induced necroptosis
Source: JCI Insight. 2020 Dec 3;5(23):e139837. doi: 10.1172/jci.insight.139837 (PMC7714407; doi:10.1172/jci.insight.139837)
Supplement: supplemental data [file jciinsight-5-139837-s079.pdf]

SUPPLEMENTAL FIGURES (Uzunparmak *et. al*.)

Figure S1. Effects of knockdown of *CASP8* on *in vitro* cell proliferation and clonogenicity in HNSCCs.

Figure S2. Effects of treatment with Birinapant and zVAD-FMK with or without TNFα on cell viability in HNSCCs under *CASP8* loss

Figure S3. Effects of treatment with Birinapant and zVAD-FMK with or without TRAIL on cell viability in HNSCCs under *CASP8* loss

Figure S4. Effects of treatment with Birinapant and zVAD-FMK on clonogenicity in HNSCCs under *CASP8* loss

Figure S5. Necroptotic effects of treatment with Birinapant and zVAD-FMK are enhanced under *CASP8* loss in HNSCCs.

Figure S6. Loss of *CASP8* increases the radiosensitizing effects of Birinapant or Birinapant plus zVAD-FMK through induction of necroptosis.

Figure S7. Loss of *CASP8* enhances radiation killing by Birinapant or Birinapant plus zVAD-FMK through induction of necroptosis.

Figure S8. Necroptosis sensitivity in HNSCC cell lines

Figure S9. Validation of *in vitro* and *in vivo CASP8* knockdown using Tetracycline-Regulated Inducible RNA interference (RNAi) system

Figure S10. Treatment schema for *in vivo* experiments

Figure S11. Inhibition of *CASP8* function with Emricasan increases radiation killing by Birinapant in HNSCCs.

Figure S12. Inhibition of *CASP8* function with Emricasan enhances radiosensitizing effects of Birinapant in HNSCCs.

Supplemental Figure 1

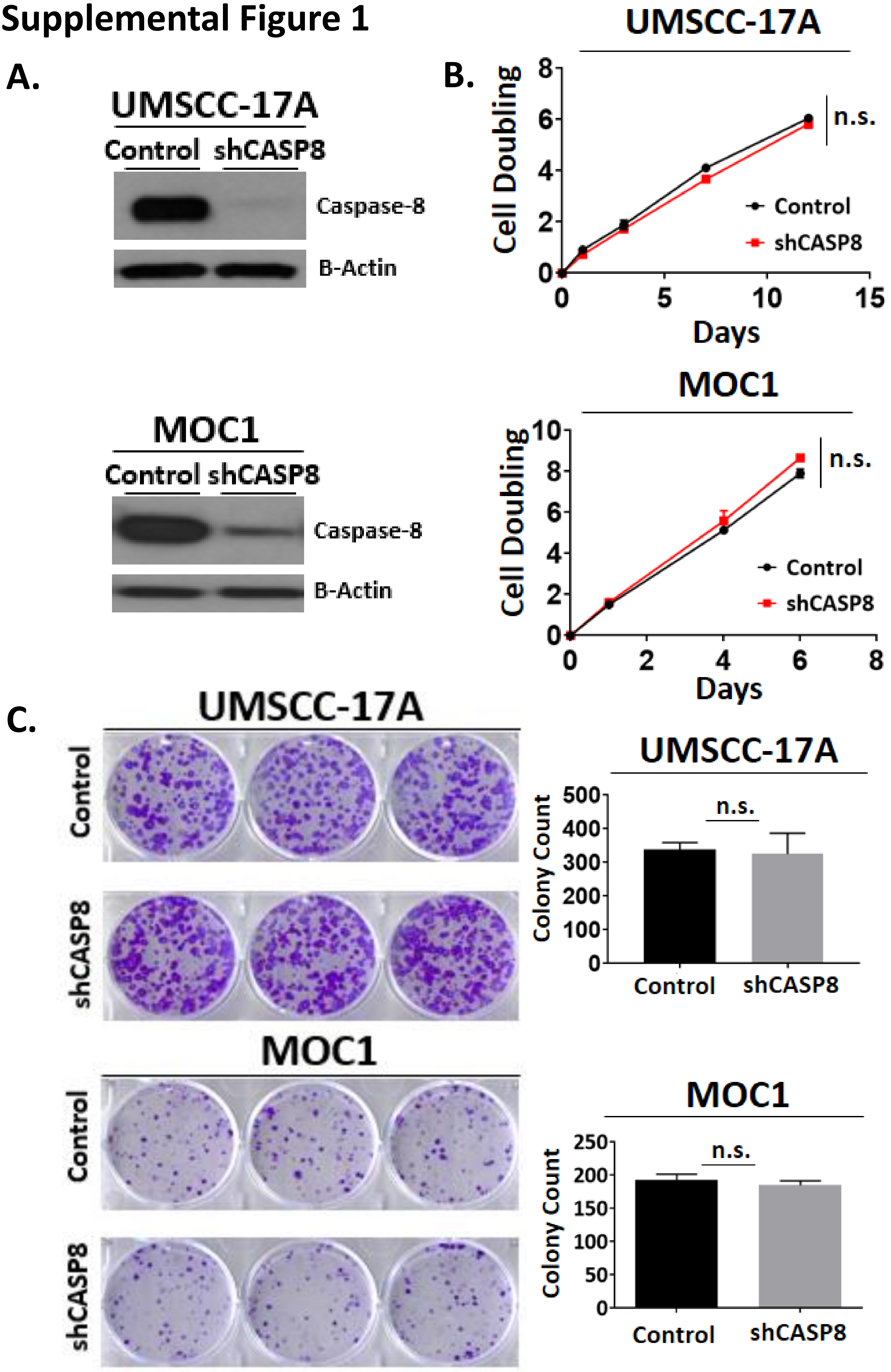

Supplemental Figure 1. Effects of knockdown of *CASP8* on *in vitro* cell proliferation and clonogenicity in HNSCCs

A. *CASP8* was knockdown using shRNA in UMSCC-17A and MOC1 HNSCC cell lines. Cell lysates obtained from the engineered control (scrambled shRNA) and shCASP8 cell lines were subjected to WB analysis for the validation of *CASP8* knockdown. β-Actin was used as loading control. **B.** Control and shCASP8 UMSCC-17A and MOC1 cell lines were subjected to cell proliferation analysis by Cell-Titer Glo. Luminescence reads were taken at the indicated time points and normalized to Day 0 reads to calculate cell doublings. Samples were run in replicates of four. 2-way ANOVA was used for statistical analysis. **C.** Control and shCASP8 UMSCC-17A and MOC1 cell lines were subjected to clonogenic survival analysis. Colony counts were used to assess baseline clonogenicity. Samples were run in triplicates. Student *t* test was used for statistics. All experiments detailed above were repeated three times with similar results.

Supplemental Figure 2

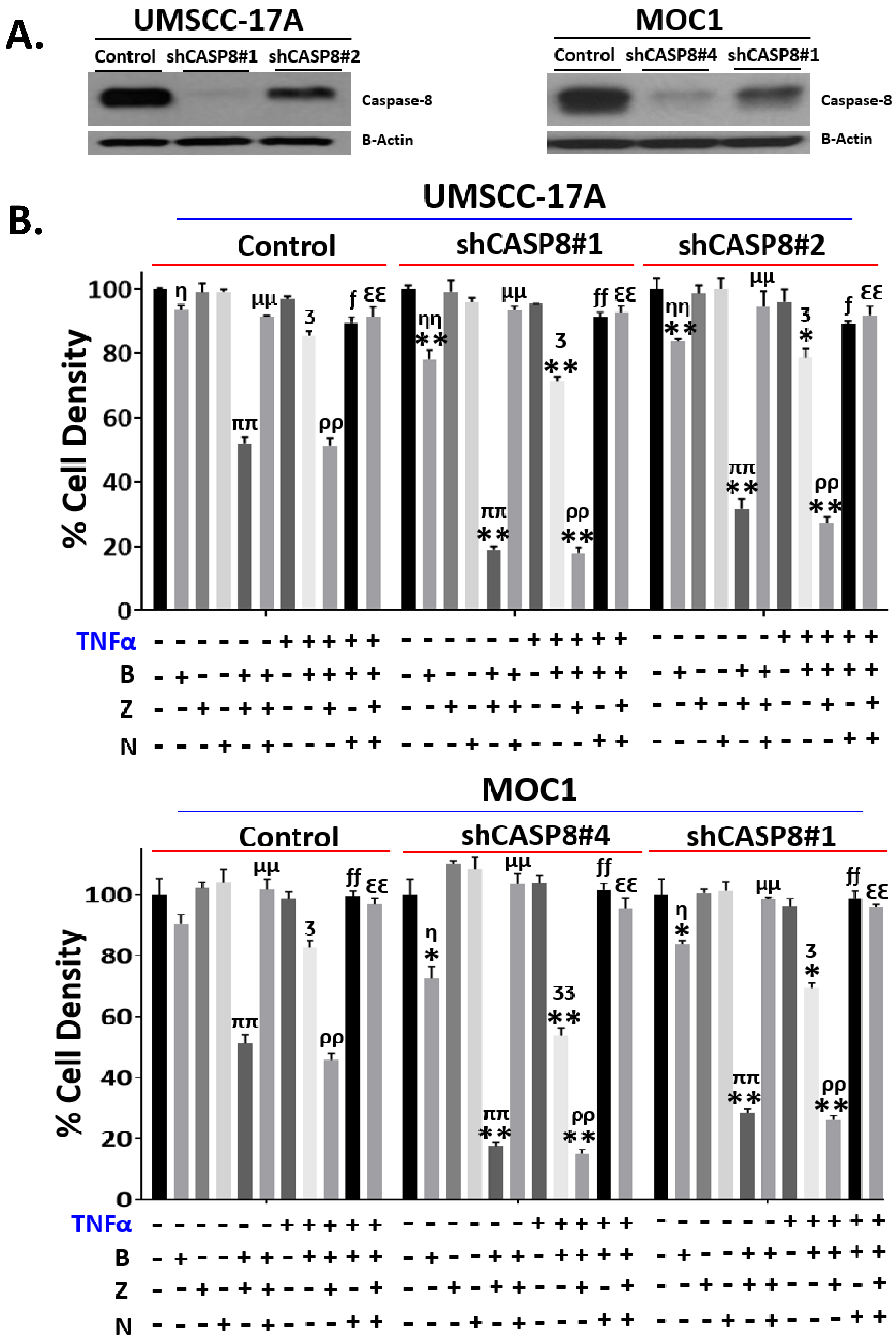

Supplemental Figure 2. Effects of treatment with Birinapant and zVAD-FMK with or without TNFα on cell viability in HNSCCs under *CASP8* loss

A. *CASP8* was knockdown using shRNA in UMSCC-17A and MOC1 HNSCC lines using 2 independent shRNA constructs. Knockdown of *CASP8* was validated by WB analysis. **B.** UMSCC-17A and MOC1 control and shRNA knockdown (shCASP8) cells were treated with Birinapant (**B** [200nmol/L for the UMSCC-17A cells; 1μmol/L for the MOC1 cells]), zVAD-FMK (**Z** [5μmol/L for both the cell lines]), Necrostatin-1s (**N** [10μmol/L for both the cell lines]) in the presence and absence of TNFα (**T** [50ng/mL for both the cell lines]) or the combinations as indicated for 24 hours. Cell viability was assessed using Cell-Titer Glo. Values normalized to nontreated cells from the same experiment to calculate % cell density. (This supplemental figure is related to **Figure 2A).** All treatments were carried out in replicates of four. Student *t* test was used for statistics. *****, *P*<0.05; ******, *P*<0.001 when comparing the effects of B, B+Z , T+B or T+B+Z conditions between isogenic control and shCASP8 cell lines. <u>The following symbols are used to make comparisons between the indicated treatment conditions for each individual cell line</u>: **η**, *P*<0.05; **ηη**, *P*<0.001 to compare no treatment (NT) vs B. **ʒ,** *P*<0.05; **ʒʒ**,*P*<0.001 to compare B vs T+B. ***ƒ***, *P*<0.05; ***ƒƒ**, P*<0.001 to compare T+B vs T+B+N. **Ɛ,** P<0.05; **ƐƐ,** *P*<0.001 to compare T+B+Z vs T+B+Z+N. **μ,** *P*<0.05; **μμ,** *P*<0.001 to compare B+Z vs B+Z+N. **π,** *P*<0.05; **ππ,** *P*<0.001 to compare B vs B+Z. **ρ,** *P*<0.05; **ρρ,** *P*<0.001 to compare T+B vs T+B+Z. All experiments detailed above were repeated three times with similar results.

Supplemental Figure 3

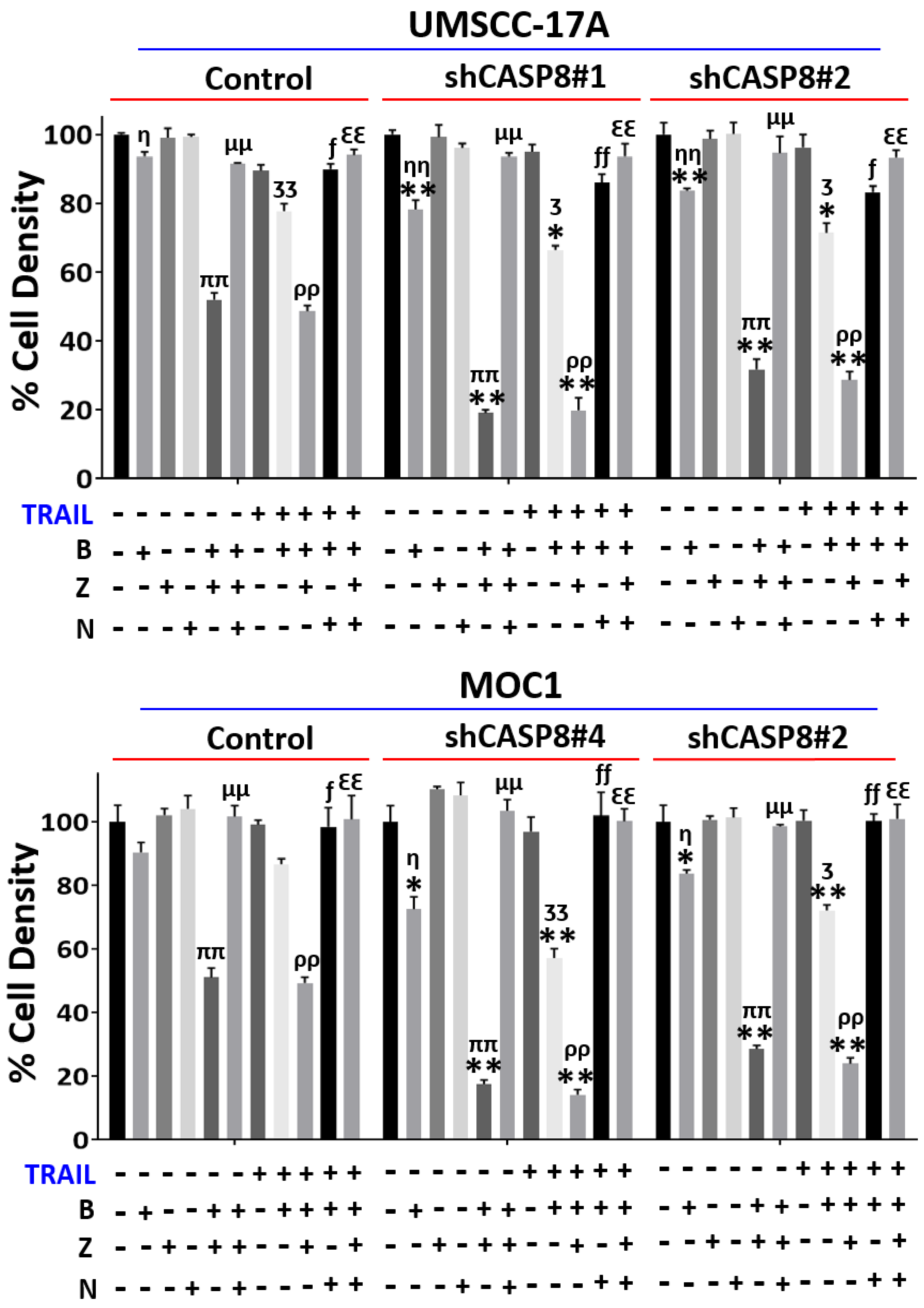

Supplemental Figure 3. Effects of treatment with Birinapant and zVAD-FMK with or without TRAIL on cell viability in HNSCCs under *CASP8* loss

UMSCC-17A and MOC1 control and shRNA knockdown (shCASP8) cells were treated with Birinapant (**B** [200nmol/L for the UMSCC-17A cells; 1µmol/L for the MOC1 cells]), zVAD-FMK (**Z** [5µmol/L for both the cell lines]), Necrostatin-1s (**N** [10µmol/L for both the cell lines]) in the presence and absence of TRAIL (**T** [10ng/ml for the UMSCC-17A cells; 50ng/mL for the MOC1 cells]) or the combinations as indicated for 24 hours. Cell viability was assessed using Cell-Titer Glo. Values normalized to nontreated cells from the same experiment to calculate % cell density. (This supplemental figure is related to **Figure 2A).** All treatments were carried out in replicates of four and experiments were repeated three times with similar results. Student *t* test was used for statistics. *****, *P*<0.05; ******, *P*<0.001 when comparing the effects of B, B+Z , T+B or T+B+Z conditions between isogenic control and shCASP8 cell lines. The following symbols are used to make comparisons between the indicated treatment conditions for each individual cell line: **η**, *P*<0.05; **ηη**, *P*<0.001 to compare no treatment (NT) vs B. **Ʒ,** *P*<0.05; **ƷƷ**,*P*<0.001 to compare B vs T+B. ***ƒ***, *P*<0.05; ***ƒƒ**, P*<0.001 to compare T+B vs T+B+N. **Ɛ,** P<0.05; **ƐƐ,** *P*<0.001 to compare T+B+Z vs T+B+Z+N. **µ,** *P*<0.05; **µµ,** *P*<0.001 to compare B+Z vs B+Z+N. **π,** *P*<0.05; **ππ,** *P*<0.001 to compare B vs B+Z. **ρ,** *P*<0.05; **ρρ,** *P*<0.001 to compare T+B vs T+B+Z.

Supplemental Figure 4

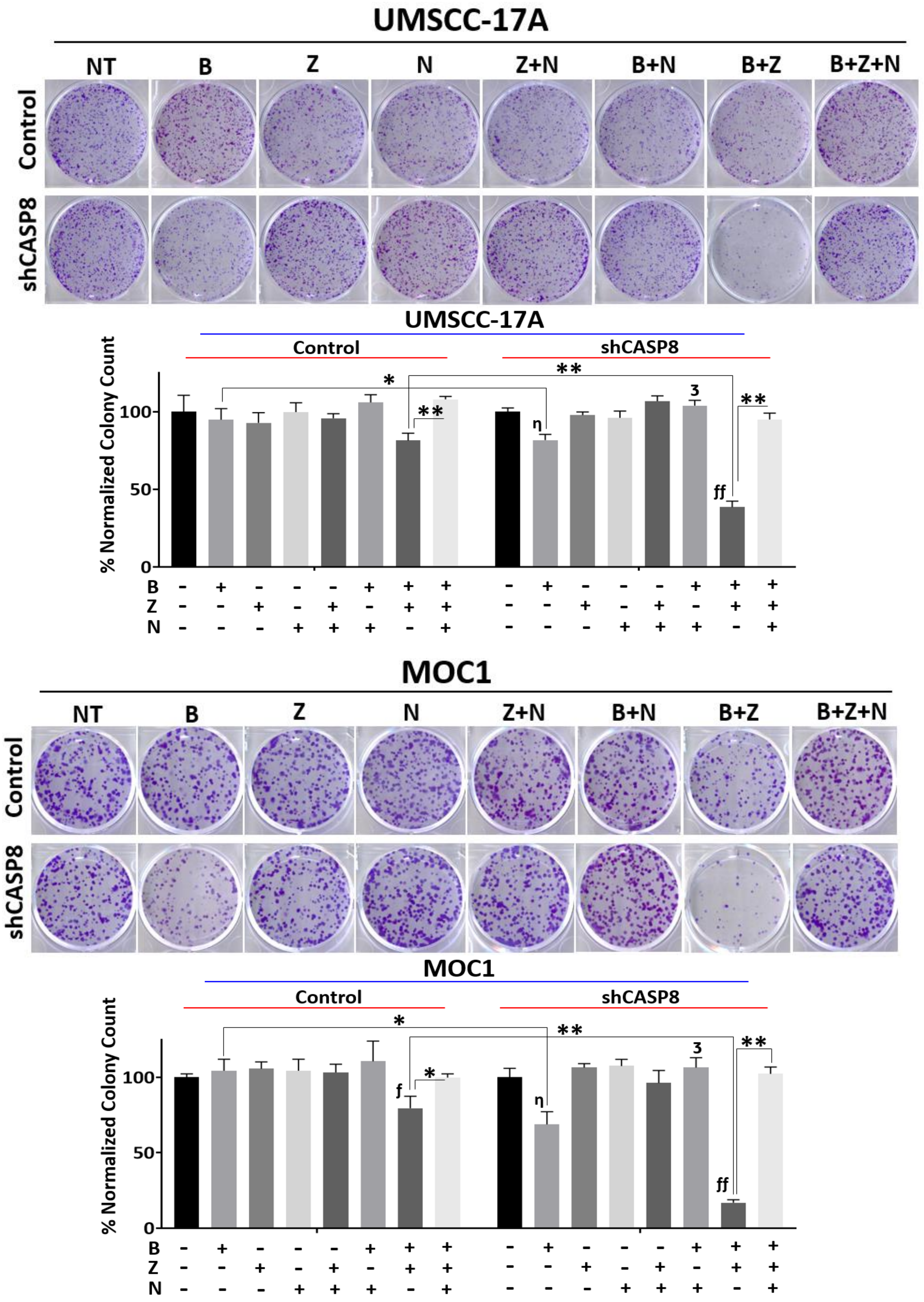

Supplemental Figure 4. Effects of treatment with Birinapant and zVAD-FMK on clonogenicity in HNSCCs under *CASP8* loss

UMSCC-17A and MOC1 control and shRNA knockdown (shCASP8) cells were treated with Birinapant (**B** [200nmol/L for the UMSCC-17A cells; 1µmol/L for the MOC1 cells]), zVAD-FMK (**Z** [5µmol/L for both the cell lines]), Necrostatin-1s (**N** [10µmol/L for both the cell lines]) or the combinations as indicated. 24 hour after treatments, drug dilutions were washed out, colonies were allowed to form for 5-12 days, after which they were stained and counted. Surviving colonies were normalized to nontreated cells from the same experiment. % normalized colony counts were plotted (This supplemental figure is related to **Figure 2B).** All treatments were carried out in triplicates and experiments were repeated three times with similar results. Student *t* test was used for statistics. *****, *P*<0.05; ******, *P*<0.001 for the indicated pairwise comparisons. The following symbols are used to make comparisons between the indicated treatment conditions for each individual cell line: **η**, *P*<0.05; **ηη**, *P*<0.001 to compare no treatment (NT) vs B. **ʒ,** *P*<0.05; **ʒʒ**, *P*<0.001 to compare B vs B+N. ***ƒ***, *P*<0.05; ***ƒƒ***, *P*<0.001 to compare B vs B+Z.

Supplemental Figure 5

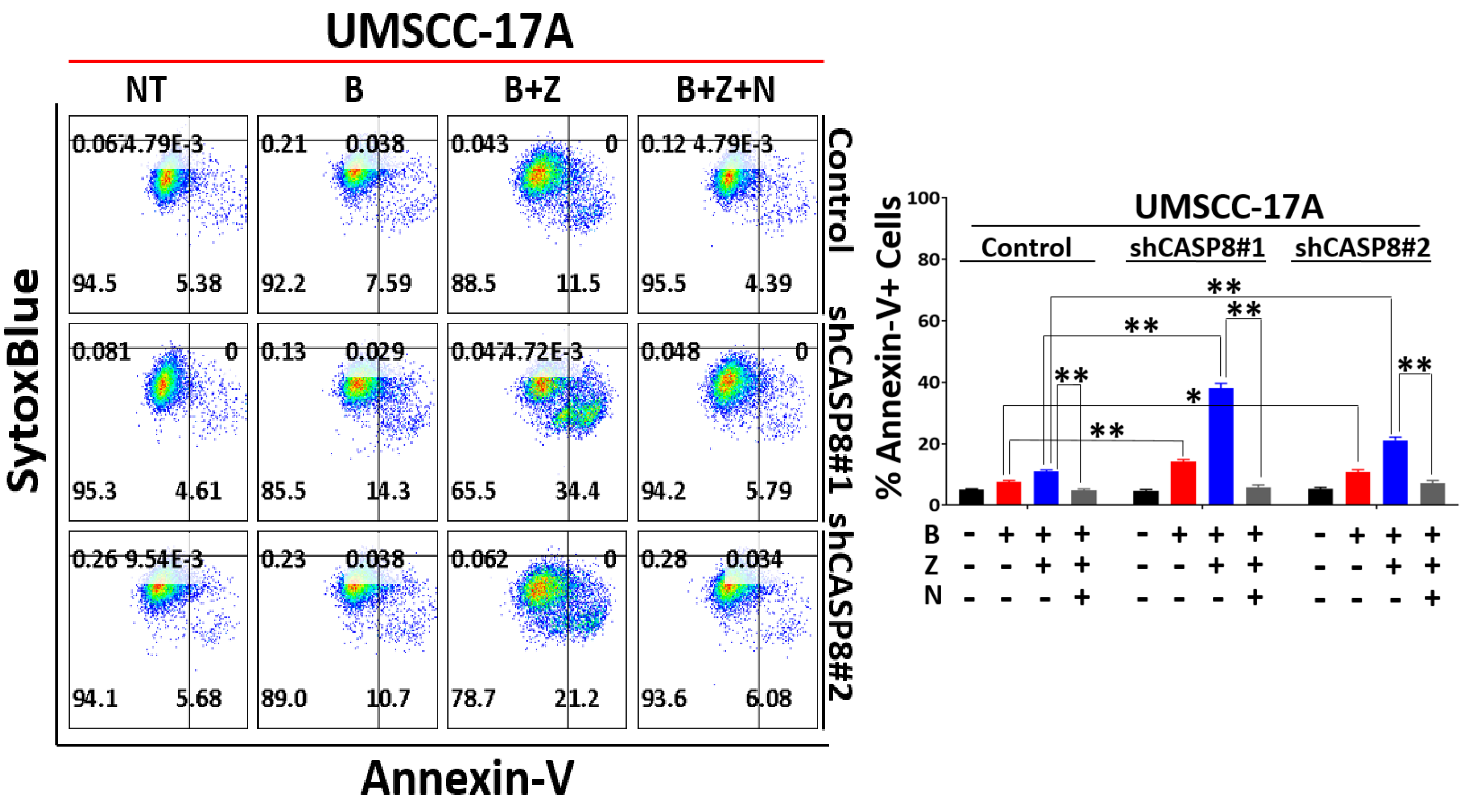

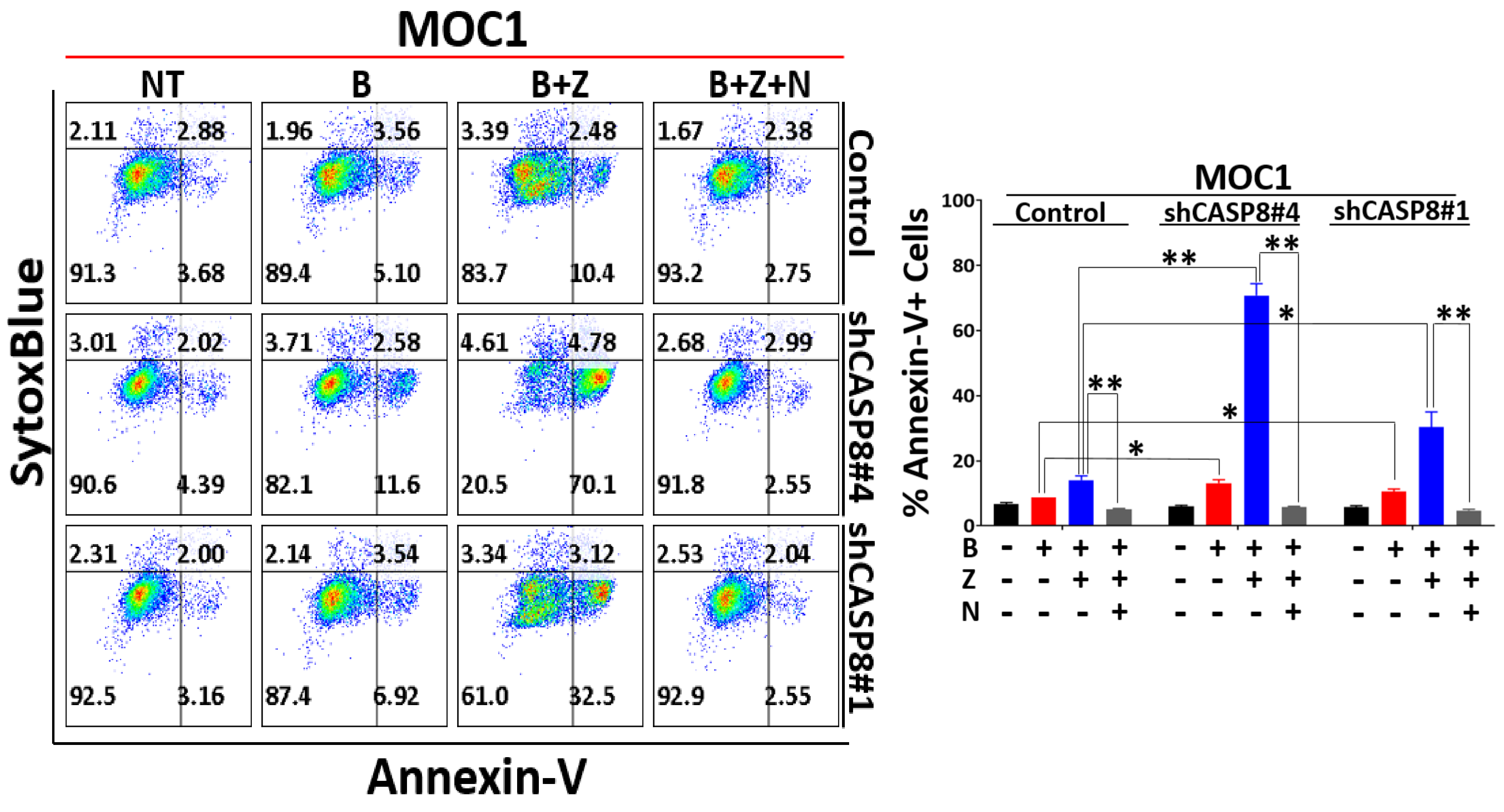

Supplemental Figure 5. Necroptotic effects of treatment with Birinapant and zVAD-FMK are enhanced under *CASP8* loss in HNSCCs.

UMSCC-17A and MOC1 control and shRNA knockdown (shCASP8) cells (2 independent shRNA clones for each cell line) were treated with Birinapant (**B** [200nmol/L for the UMSCC-17A cells; 1μmol/L for the MOC1 cells]), zVAD-FMK (**Z** [5μmol/L for both the cell lines]), Necrostatin-1s (**N** [10μmol/L for both the cell lines]) or the combinations as indicated. AnnexinV-APC/SytoxBlue staining was performed 24 hour after treatments. % Annexin-V positivity was used as a measure to assess cell death (This supplemental figure is related to **Figure 2C**). All treatments were carried out in triplicates and experiments were repeated three times with similar results. Student *t* test was used for statistics. *, $P<0.05$; **, $P<0.001$ for the indicated pairwise comparisons.

Supplemental Figure 6

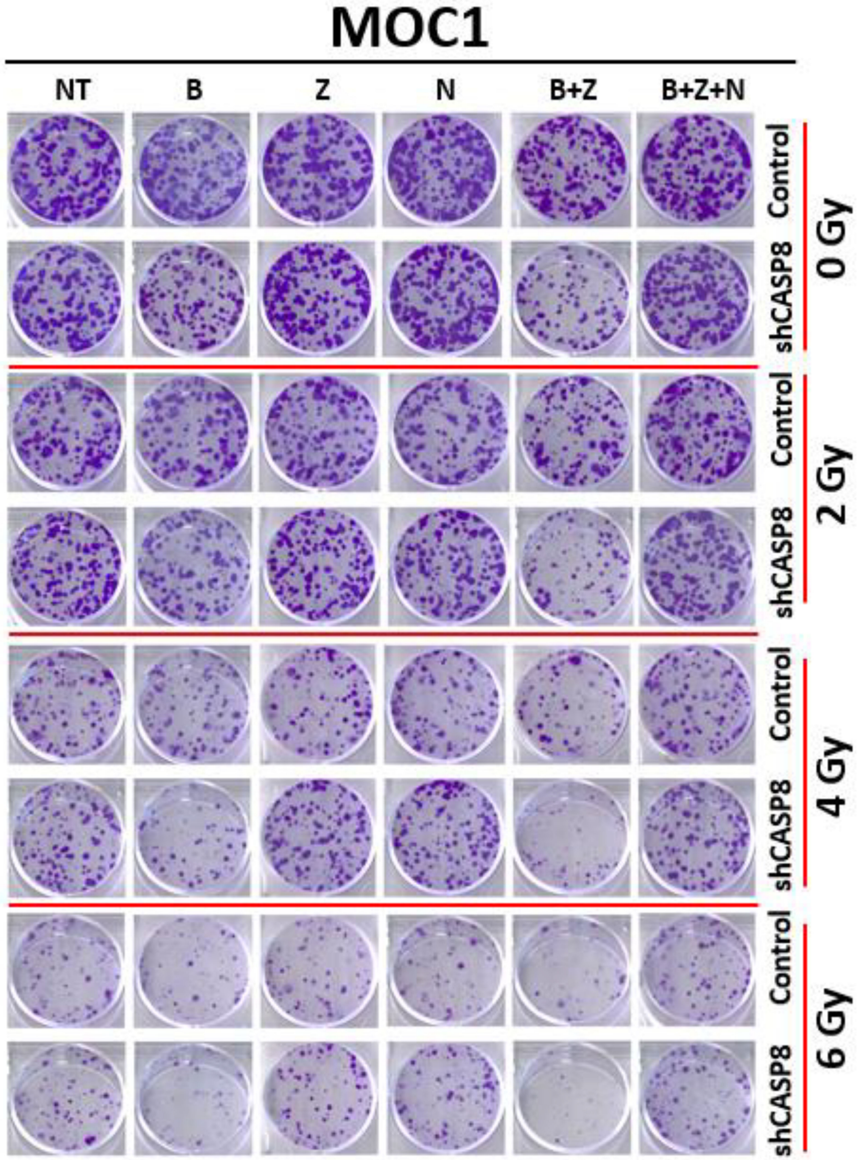

Dose Enhancement Ratio (DER) values for the cell lines		
Treatment Conditions	MOC1 Control	MOC1 shCASP8
B	1.07	1.53
Z	1.13	1.12
N	1.06	0.94
B+Z	1.24	1.81
B+Z+N	0.95	0.96

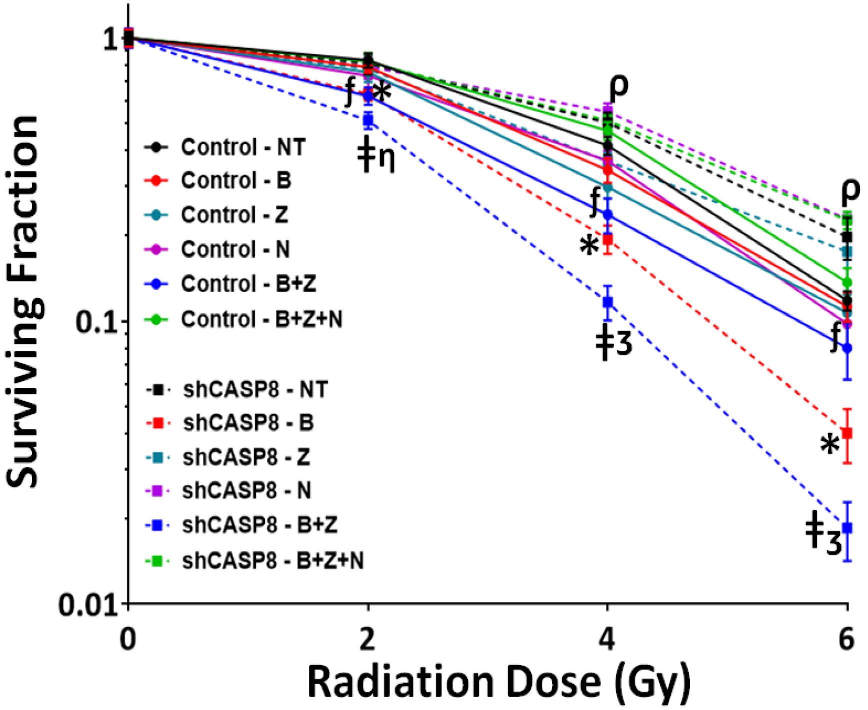

Supplemental Figure 6. Loss of *CASP8* increases the radiosensitizing effects of Birinapant or Birinapant plus zVAD-FMK through induction of necroptosis.

MOC1 control and shCASP8 cells were treated with radiation (**X** [2, 4 and 6 Gy]), Birinapant (**B** [250nmol/L]), zVAD-FMK (**Z** [5μmol/L]), Necrostatin-1s (**N** [10μmol/L]) or the combinations as indicated. 24 hour after treatments, drug dilutions were washed out, colonies were allowed to form for 5 days, after which they were stained and counted. Surviving colony counts were normalized to nontreated cells (cells treated with no drugs) of each radiation dose from the same experiment. *Log10* of surviving fractions were plotted (This supplemental figure is related to **Figure 3A**). All treatments were carried out in triplicates and experiments were repeated three times with similar results. Student *t* test was used for statistics. *, $P<0.05$; when comparing surviving fractions following X+B treatments between control and shCASP8 cells **ǂ,** $P<0.05$; when comparing surviving fractions following X+B+Z treatments between control and shCASP8 cells ***ƒ*,** $P<0.05$; when showing reversal of death upon addition of N to X+B+Z for the control cells, **η,** $P<0.05$; when showing reversal of death upon addition of N to X+B+Z for the shCASP8 cells, **ʒ,** $P<0.001$; when showing reversal of death upon addition of N to X+B+Z for the shCASP8 cells. Symbols are placed at the radiation doses they refer to. A more detailed version of the Dose Enhancement Ratio (DER) table can be found in **Supplemental Table-6.**

Supplemental Figure 7

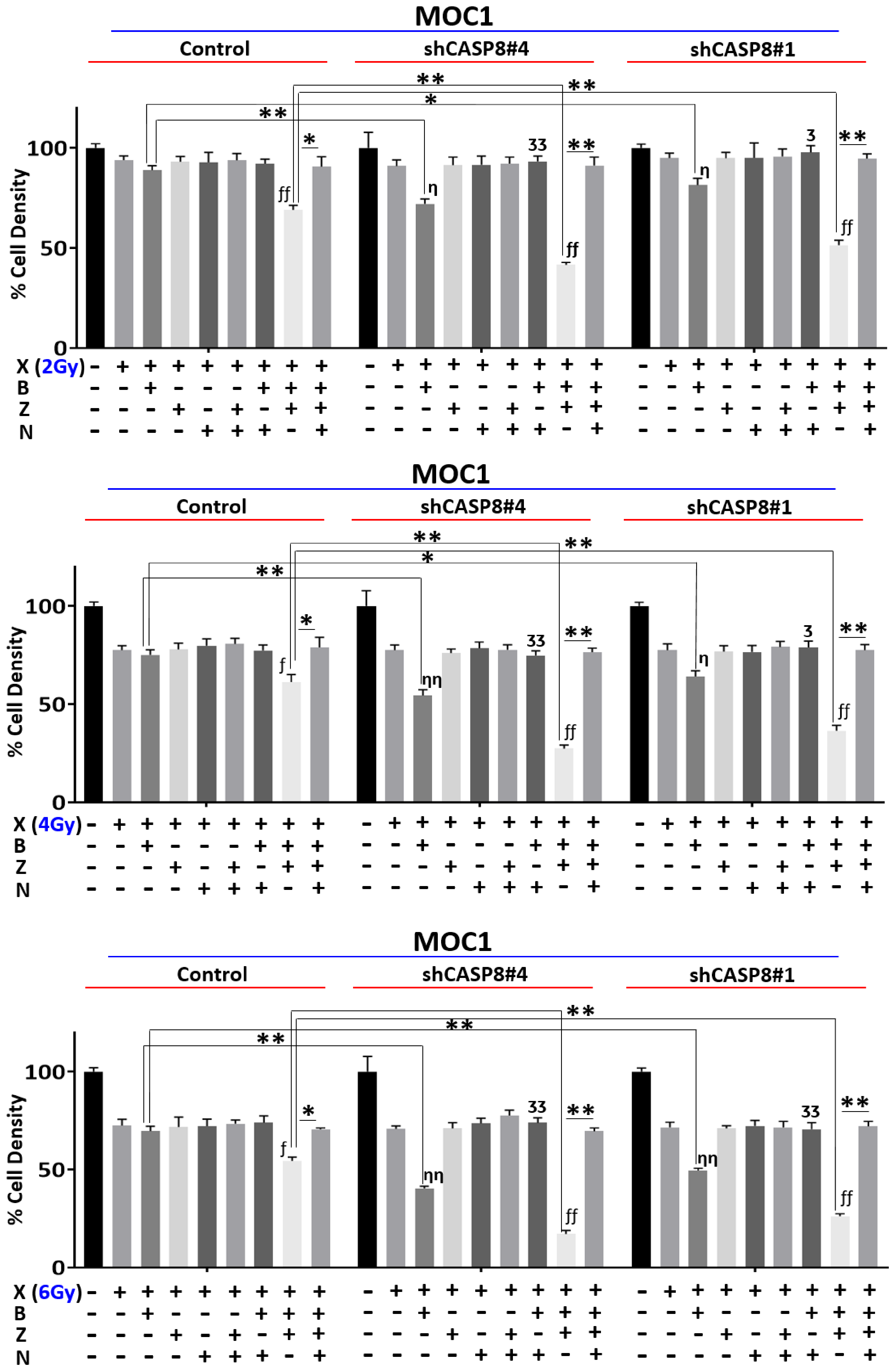

Supplemental Figure 7. Loss of *CASP8* enhances radiation killing by Birinapant or Birinapant plus zVAD-FMK through induction of necroptosis.

MOC1 control and shCASP8 cells were treated with radiation (**X** [2, 4 and 6 Gy]), Birinapant (**B** [250nmol/L]), zVAD-FMK (**Z** [5μmol/L]), Necrostatin-1s (**N** [10μmol/L]) or the combinations as indicated. 24 hour after treatments, cell viability was assessed by Cell-Titer Glo. Values normalized to nontreated cells from the same experiment to calculate % cell density (This supplemental figure is related to **Figure 3B**). All treatments were carried out in triplicates and experiments were repeated three times with similar results. Student *t* test was used for statistics. *, P<0.05; **, P<0.001 for the indicated pairwise comparisons. <u>The following symbols are used to make comparisons between the indicated treatment conditions for each individual cell line</u>: **η**, P<0.05; **ηη,** P<0.001 to compare X vs X+B. **ʒ,** P<0.05; **ʒʒ,** P<0.001 to compare X+B vs X+B+N. ***ƒ*,** P<0.05; ***ƒƒ*,** P<0.001 to compare X vs X+B+Z.

Supplemental Figure 8

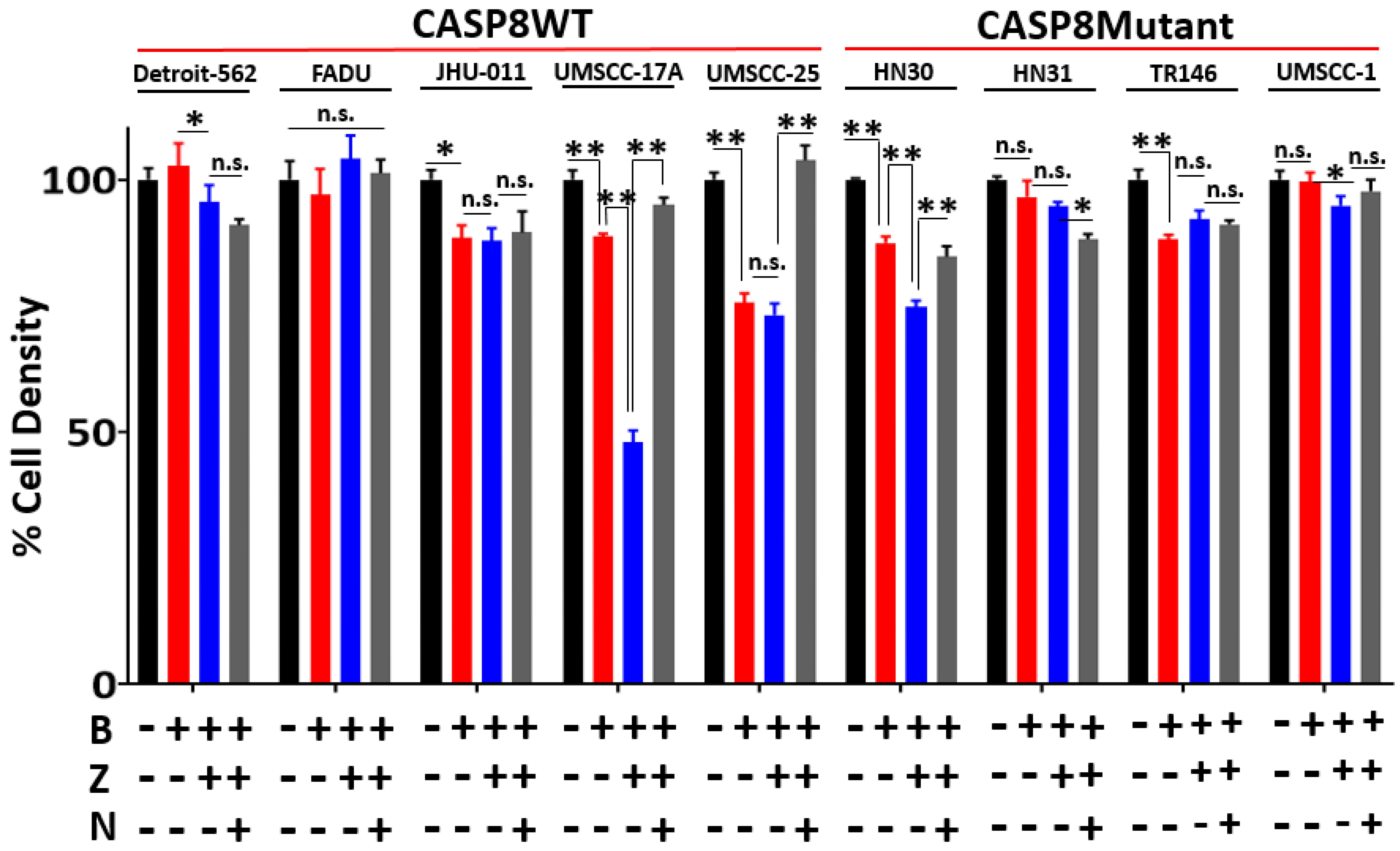

Supplemental Figure 8. Necroptosis sensitivity in HNSCC cell lines A panel of **9** human-derived HNSCC cell lines, **5** *CASP8*WT (Detroit-562, FADU, JHU-011, UMSCC-17A and UMSCC-25) and **4** *CASP8* mutant (HN30, HN31, TR146 and UMSCC-1) were treated with Birinapant (**B** [1μmol/L]), zVAD-FMK (**Z** [5μmol/L]), Necrostatin-1s (**N** [10μmol/L]) or the combinations. 24 hour after treatments, cell viability was assessed by Cell-Titer Glo. Values normalized to nontreated cells from the same experiment to calculate % cell density (This supplemental figure is related to **Figure 5A**). All treatments were carried out in triplicates and experiments were repeated three times with similar results. Student *t* test was used for statistics. *, $P<0.05$; **, $P<0.001$ for the indicated pairwise comparisons.

Supplemental Figure 9

A.

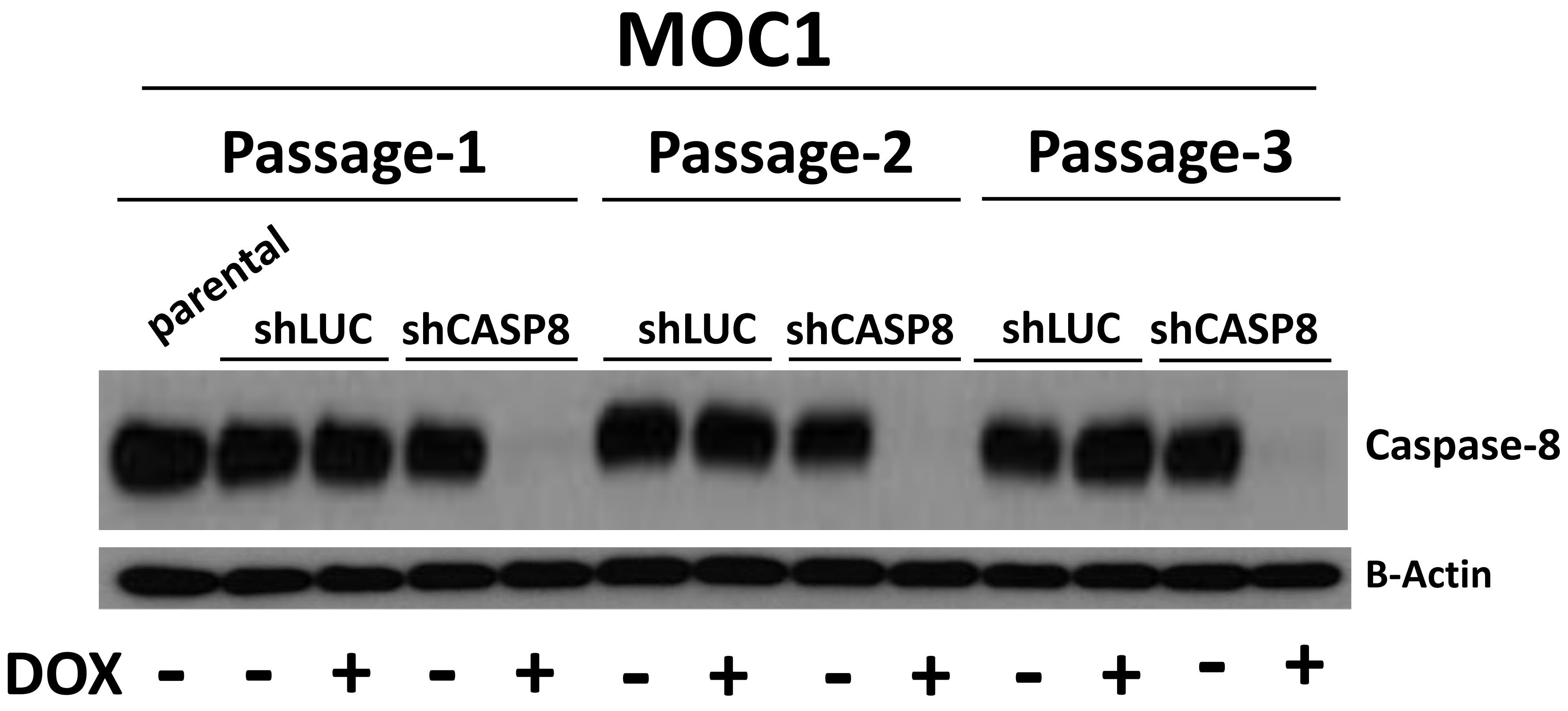

B.

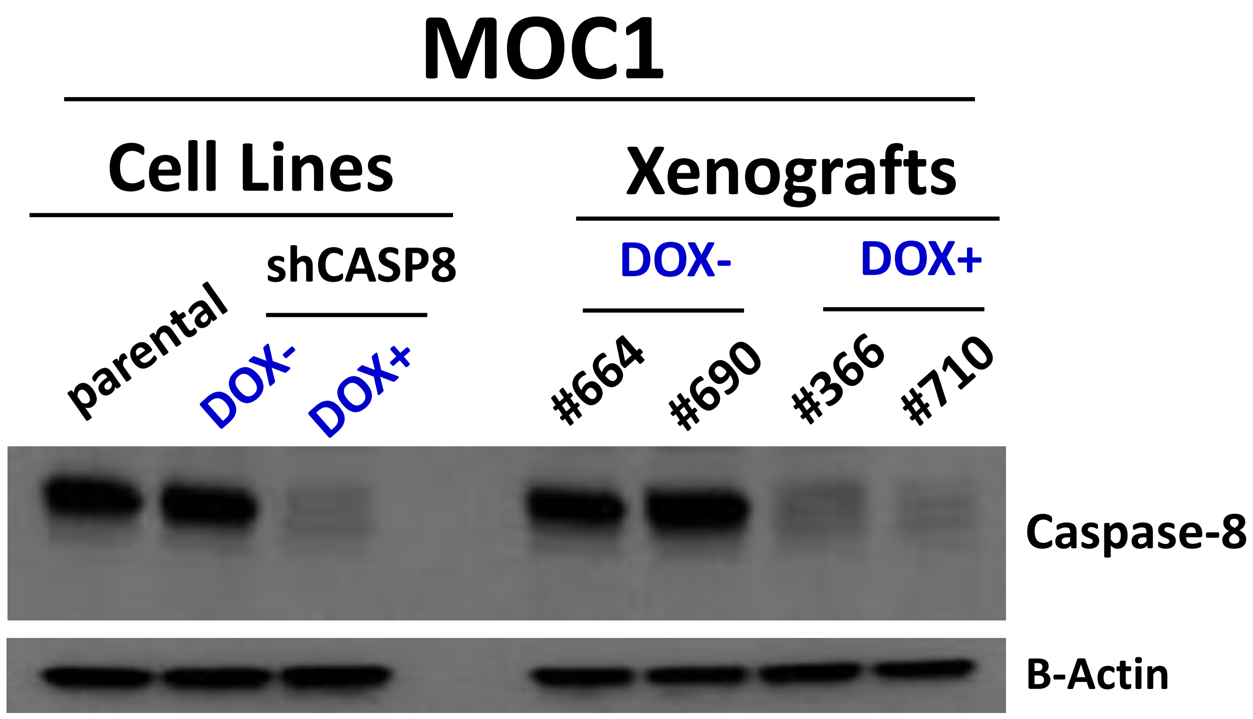

Supplemental Figure 9. Validation of *in vitro* and *in vivo CASP8* knockdown using Tetracycline-Regulated Inducible RNA interference (RNAi) system

This supplemental figure is related to **Figures 6A-B.** **A.** MOC1 cells were transduced with lentiviral constructs designed against Luciferase (shLUC) and *Casp8 (*shCASP8*)*. The engineered shLUC and shCASP8 cells were cultured in the presence and absence of Doxycycline (25ng/ml). 72 hours after treatment, cells were passaged and cell lysates were obtained. This cycle was repeated 3 times. Cell lysates obtained from each cycle along with that from MOC1 parental cells were subjected to WB analysis for Caspase-8. β-Actin was used as loading control. **B.** Tumor samples collected from control (#664, #690) and Doxycycline (DOX)-fed (#366, #710) mice were minced and cultured in medium for 48 hours. Cells shed from the indicated xenografts that have attached to culture dishes were collected and lysed. Cell lysates obtained from the tumor samples along with those from the indicated cell lines were subjected to WB analysis for Caspase-8. β-Actin was used as loading control.

Supplemental Figure 10

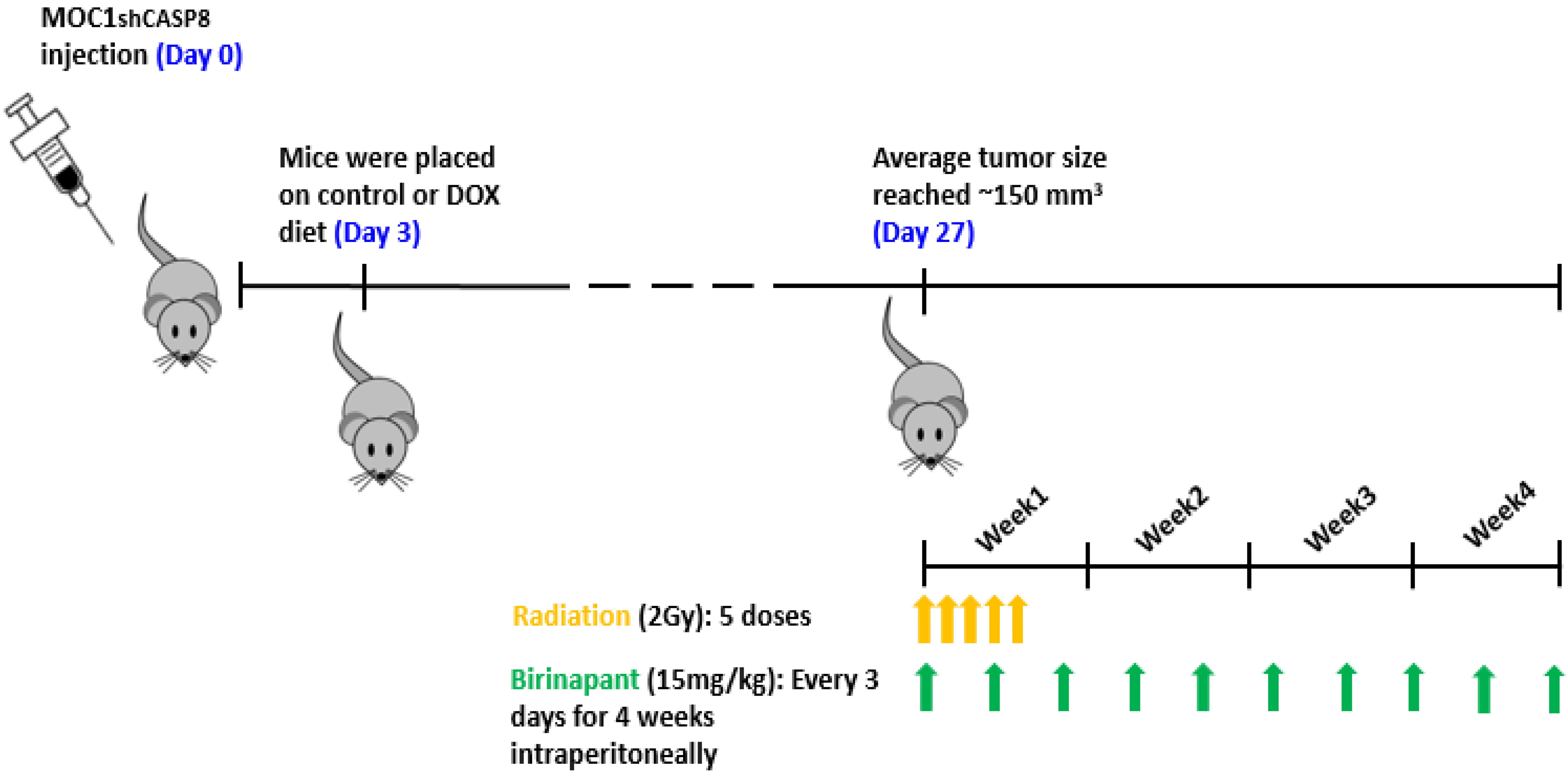

Supplemental Figure 10. Treatment schema for *in vivo* experiments

This supplemental figure is related to **Figures 6A-B.**

Procedure shown on the treatment schema: 2×10^6 MOC1 cells transduced with an inducible shRNA against *CASP8* were injected into the right flank of WT female C57BL/6 mice. Mice were randomized and placed on control or DOX diet (doxycycline hyclate added at 625 mg/kg) 3 days post injection to induce knockdown of *CASP8 in vivo* (Please refer to **Supplemental Figure 9** for the WB images). Control and *CASP8* knockdown mice were randomized into 4 treatment groups (vehicle control, 15mg/kg Birinapant, 5X2Gy radiation or combination, *n*=7-10/each) 27 days post inoculation when the average tumor volume reached ~150 mm^3. Radiation started on Day 27: 2Gy of radiation given Monday to Friday for 1 week. Birinapant started on Day 27: 15mg/kg Birinapant given intraperitoneally every 3 days for 4 weeks.

Supplemental Figure 11

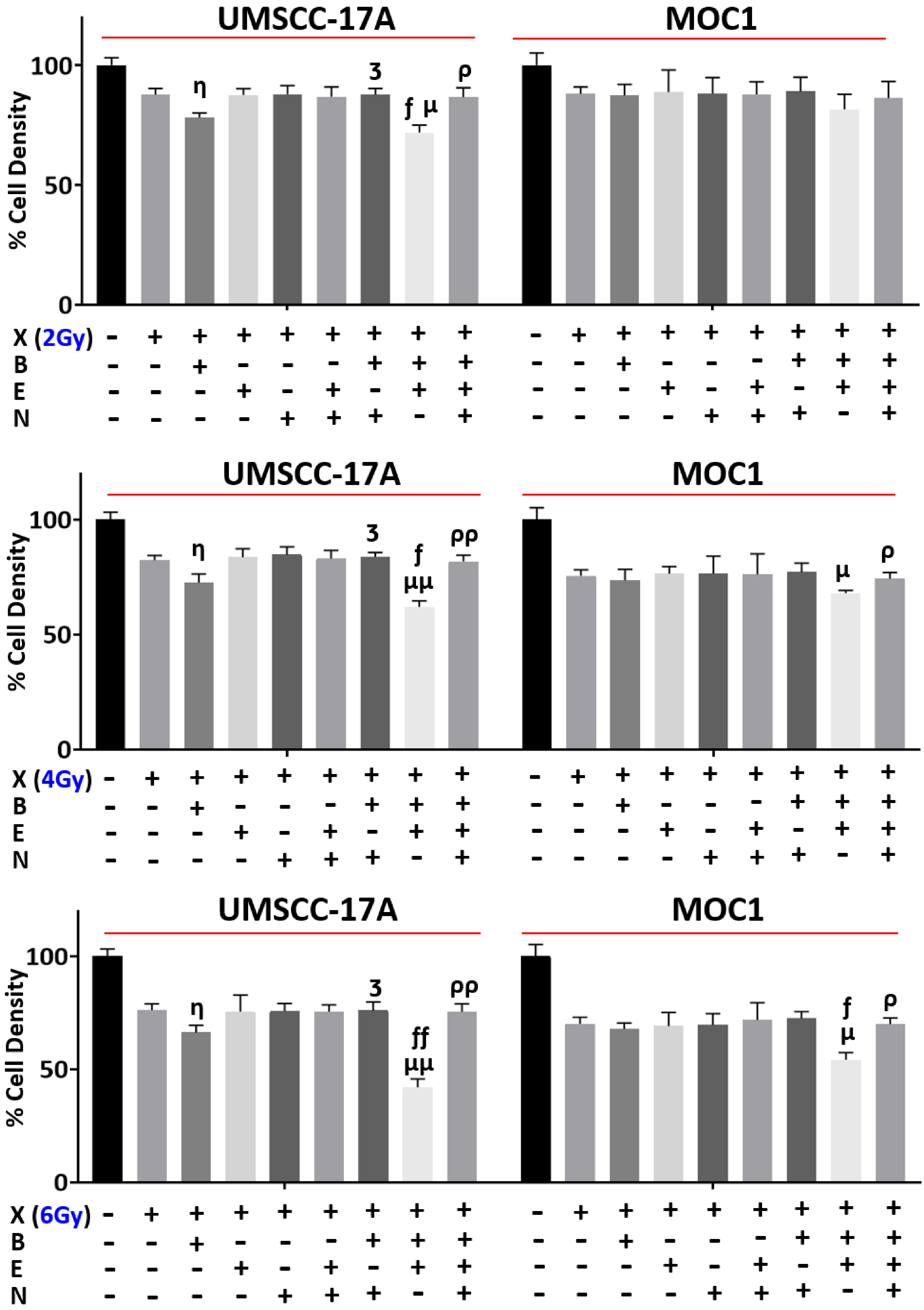

Supplemental Figure 11. Inhibition of *CASP8* function with Emricasan increases radiation killing by Birinapant in HNSCCs.

MOC1 and UMSCC-17A parental cells were treated with radiation (**X** [2, 4 and 6 Gy for both the cell lines]), Birinapant (**B** [200nmol/L for the UMSCC-17A cells and 1μmol/L for the MOC1 cells]), Emricasan (**E** [1μmol/L for both the cell lines]), Necrostatin-1s (**N** [10μmol/L for both the cell lines]) or the combinations as indicated. 24 hour after treatments, cell viability was assessed by Cell-Titer Glo. Values normalized to nontreated cells from the same experiment to calculate % cell density (This supplemental figure is related to **Figure 6C**). All treatments were carried out in replicates of four and experiments were repeated three times with similar results. Student *t* test was used for statistics. The following symbols are used to make comparisons between the indicated treatment conditions for each individual cell line: **η**, P<0.05; **ηη,** P<0.001 to compare X vs X+B. **ʒ,** P<0.05; **ʒʒ,** P<0.001 to compare X+B vs X+B+N. ***ƒ*,** P<0.05; ***ƒƒ*,** P<0.001 to compare X+B vs X+B+E. **μ,** P<0.05; **μμ,** P<0.001 X vs X+B+E. **ρ,** P<0.05; **ρρ,** P<0.001 to compare X+B+E vs X+B+E+N.

Supplemental Figure 12

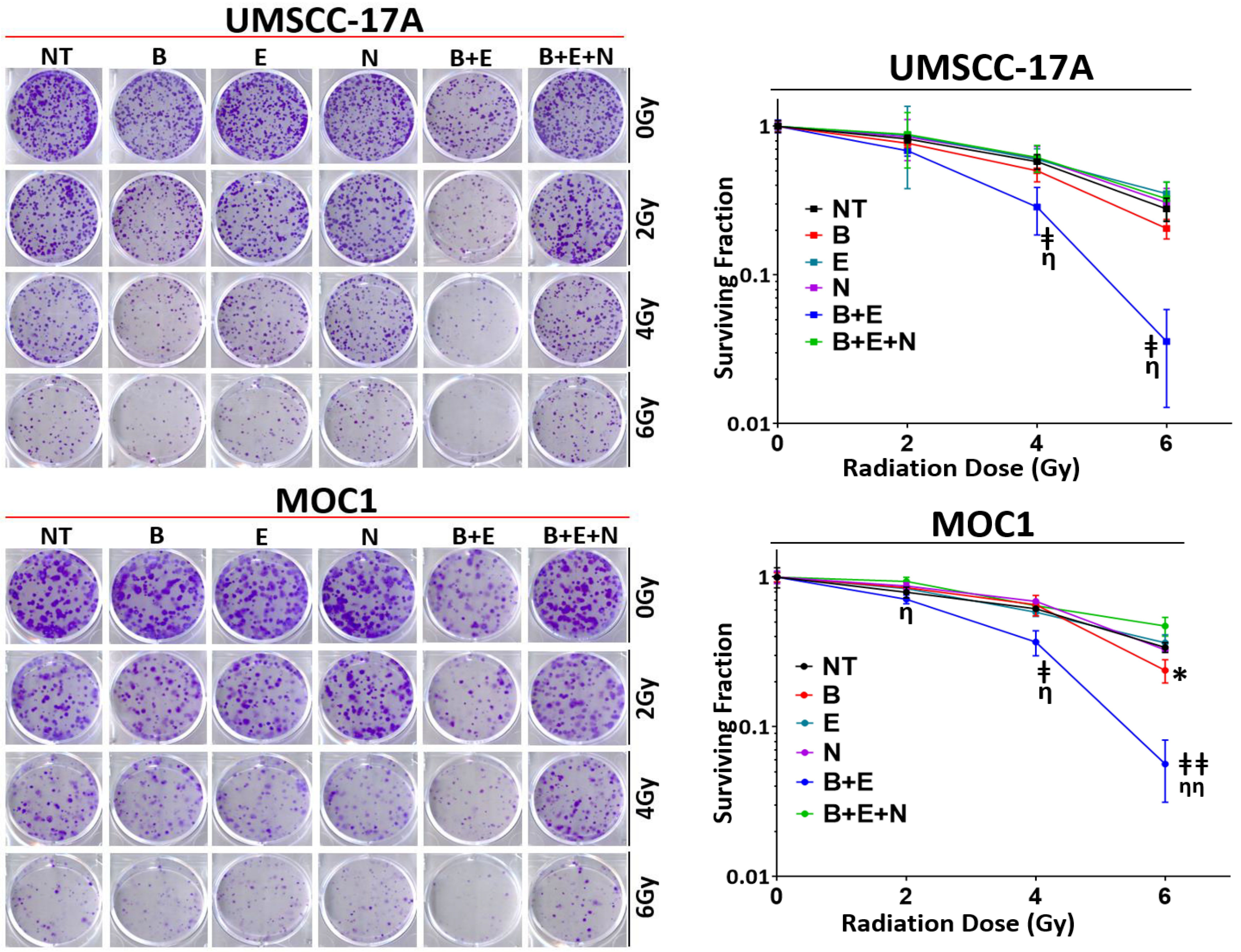

Supplemental Figure 12. Inhibition of *CASP8* function with Emricasan enhances radiosensitizing effects of Birinapant in HNSCCs.

MOC1 and UMSCC-17A parental cells were treated with radiation (**X** [2, 4 and 6 Gy for both the cell lines]), Birinapant (**B** [200nmol/L for the UMSCC-17A cells and 1μmol/L for the MOC1 cells]), Emricasan (**E** [1μmol/L for both the cell lines]), Necrostatin-1s (**N** [10μmol/L for both the cell lines]) or the combinations as indicated. 24 hour after treatments, drug dilutions were washed out, colonies were allowed to form for 5-12 days, after which they were stained and counted. Surviving colony counts were normalized to nontreated cells (cells treated with no drugs) of each radiation dose from the same experiment. Log10 of surviving fractions were plotted (This supplemental figure is related to **Figure 6D**). All treatments were carried out in triplicates and experiments were repeated three times with similar results. Student *t* test was used for statistics. *, P<0.05; when comparing X+B to X alone for the indicated radiation dose. **ǂ,** *P*<0.05 and **ǂǂ,** *P*<0.001; when comparing X+B+E to X alone for the indicated radiation doses. **η,** *P*<0.05 and **ηη,** *P*<0.001; when comparing X+B+E to X+B+E+N for the indicated radiation doses.

SUPPLEMENTAL TABLES (Uzunparmak *et. al*.)

Table S1. Univariate and multivariate Cox-regression analyses of selected factors for overall survival (OS) in HPV-negative oral cancers

Table S2. HNSCC cell lines used in the study

Table S3. shRNA and CRIPSR-Cas9 sgRNA oligo sequences

Table S4. List of reagents/drugs used in the study

Table S5. List of antibodies used for western blotting

Table S6. Dose enhancement ratio (DER) values for the cell lines used in Figure 3A and Supplemental Figure 6

Table S7. *p* values for key pairwise comparisons in mouse tumor growth analysis

Table S8. *p* values for key pairwise comparisons in mouse survival analysis

Table S9. Key tumor growth parameters assessed in the mouse study presented in Figure 6

Supplemental Table 1: Univariate and multivariate Cox-regression analyses of selected factors for overall survival (OS) in HPV-negative oral cancers

**Univariate and Multivariate Cox-Regression Analysis**							
		**Univariate**			**Multivariate**		
**Variable**		**HR**	**CI95%**	**Wald p-value**	**HR**	**CI95%**	**Wald p-value**
CASP8 / *HRAS* status							
	CASP8 WT or *HRAS* Mut		ref	**0.023**		ref	**0.015**
	CASP8 Mut and *HRAS* WT	0.56	0.34-0.93		0.47	0.26-0.87	
Age							
	continuous	1.02	1-1.03	**0.020**	1.01	0.89-1.03	0.572
Neoplasm Histologic Grade							
	G1		ref			ref	
	G2	0.62	0.35-1.1	0.089	0.59	0.29-1.21	0.116
	G3	0.49	0.25-0.92		0.43	0.19-0.96	
Anatomic subdivision							
	Oral tongue		ref				
	Oral cavity	0.86	0.54-1.38				
	Lip	1.07	0.14-7.79				
	Hard palate	1.45	0.35-6.02	0.690			
	Floor of the mouth	0.67	0.42-1.07				
	Buccal mucosa	0.92	0.44-1.88				
	Alveolar ridge	1.2	0.47-3.05				
Tumor stage							
	I		ref			ref	
	II	0.6	0.17-2.06		1.26	0.32-4.9	
	III	0.46	0.13-1.57	**0.004**	0.51	0.14-1.79	**<0.0001**
	IVA	0.27	0.08-0.86		0.16	0.05-0.53	
	IVB	0.17	0.04-0.77		0.06	0.01-0.34	
Radiation therapy							
	No		ref	**0.029**		ref	**<0.0001**
	Yes	1.55	1.04-2.31		3.57	2.19-5.83	

Multivariate model included the variables: CASP8 / HRAS status; Age; Neoplasm Histologic Grade; Tumor Stage; and Radiation therapy. HR = Hazard ratio; CI95% = confidence interval at 95%

Supplemental Table 2: HNSCC cell lines used in the study

Cell Line	Culture Media	Primary Source
Detroit-562	DMEM, 10% FBS	ATCC
FADU	DMEM, 10% FBS	ATCC
HN30	DMEM, 10% FBS	Dr. John Ensley, Wayne State University
HN31	DMEM, 10% FBS	Dr. John Ensley, Wayne State University
JHU-011	RPMI 1640, 10% FBS	Dr. David Sidransky, Johns Hopkins University
TR146	DMEM, 10% FBS	Dr. Thomas Rupniak, Imperial Cancer Research Fund, London
UMSCC-1	DMEM, 10% FBS	Dr. Thomas E. Carey, University of Michigan
UMSCC-17A	DMEM, 10% FBS	Dr. Thomas E. Carey, University of Michigan
UMSCC-25	DMEM, 10% FBS	Dr. Thomas E. Carey, University of Michigan
MOC1	IMDM, 5% FBS	Dr Ravindra Uppaluri, Washington University School of Medicine

Supplemental Table 3: shRNA and CRIPSR-Cas9 sgRNA oligo sequences

**mouse *Casp8* CRISPR-Cas9 sgRNA sequences**	**DNA Sequence (5' to 3')**
Casp8 for-1	CACCGGTGTCGTCTATGGAACGGAT
Casp8 rev-1	AAACATCCGTTCCATAGACGACACC
Casp8 for-2	CACCGCAAGAACTATATTCCGGATG
Casp8 rev-2	AAACCATCCGGAATATAGTTCTTGC
Casp8 for-3	CACCGTAGCTTCTGGGCATCCTCGA
Casp8 rev-3	AAACTCGAGGATGCCCAGAAGCTAC
**human *CASP8* shRNA oligo sequences**	
CASP8 shRNA#1 (shCASP8#1)	TAATTCGGAAGAGCAGCTC
CASP8 shRNA#1 (shCASP8#2)	TTCCTTCTCCCAGGATGAC
**mouse *Casp8* shRNA oligo sequences**	
Casp8 shRNA#1 (shCASP8#1)	AAACTTTGTCTGAAGTCCG
Casp8 shRNA#4 (shCASP8#4)	TTTCATTTGCAGTGCAGTC
**mouse *Casp8* inducible shRNA oligo sequences**	
Casp8 inducible shRNA#10 (shCASP8)	TTTCATTTGCAGTGCAGTC
**Luciferase inducible shRNA oligo sequences**	
luciferase inducible shRNA (shLUC)	CGCTGAGTACTTCGAAATGTC
**mouse *Ripk3* shRNA oligo sequences**	
Ripk3 shRNA (shRIP3)	TACCTCGGAGACAGCAGCA

Supplemental Table 4: List of reagents/drugs used in the study

No.	Reagent/drug name	Catalog number	Company
1	Birinapant	S7015	Selleckchem
2	zVAD-FMK	FMK001	R&D Systems
3	human recombinant TNFα	210-TA	R&D Systems
4	human recombinant TRAIL	375-TEC	R&D Systems
5	murine recombinant TNFα	315-01A	Peprotech
6	murine recombinant TRAIL	315-19	Peprotech
7	Necrostatin-1s (7-Cl-O-Nec1)	10-4544	Focus Biomolecules
8	Puromycin dihydrochloride from Streptomyces alboniger	P8833	Sigma-Aldrich
9	Doxycycline	631311	Takara Bio USA

Supplemental Table 5: List of antibodies used for western blotting

No.	Antibody name	Catalog number	Source	Company	Dilution
1	human CASP8	551242	mouse	BD Biosciences	1:1000
2	human/mouse CASP8	AF1650	rabbit	R&D Systems	1:400
3	RIP1	610458	mouse	BD Biosciences	1:1000
4	human RIP3 (E1Z1D)	13526	rabbit	Cell Signaling	1:1000
5	mouse RIP3 (D4G2A)	95702	rabbit	Cell Signaling	1:1000
6	MLKL (D216N)	14993	rabbit	Cell Signaling	1:1000
7	MLKL (mouse specific)	28640	rabbit	Cell Signaling	1:1000
8	phospho-RIP1 (S166)	31122	rabbit	Cell Signaling	1:1000
9	phospho-MLKL (S358) (D6H3V)	91689	rabbit	Cell Signaling	1:1000
10	PARP	9542	rabbit	Cell Signaling	1:1000
11	Anti-HA	H3663	mouse	Sigma-Aldrich	1:2000
12	β-actin	A1978	mouse	Sigma-Aldrich	1:10000
13	Anti-rabbit IgG	1706515	goat	BioRad	1:5000
14	Anti-mouse IgG	1706516	goat	BioRad	1:5000

Supplemental Table 6: Dose enhancement ratio (DER) values for the cell lines used in Figure 3A and Supplemental Figure 6

**Dose Enhancement Ratio (DER) values for the cell lines used**		
	**MOC1 Control**	**MOC1 shCasp8**
**Treatment Conditions**		
B	1.07	1.53
Z	1.13	1.12
N	1.06	0.94
B+Z	1.24	1.81
B+Z+N	0.95	0.96

B: Birinapant; **Z:** zVAD FMK; **N:** Necrostatin 1s; **$SF_{0.25}$:** Surviving fraction of 0.25;

DER = Mean radiation dose to reach $SF_{0.25}$ for control/Mean radiation dose to reach $SF_{0.25}$ for each of the given treatments

DER>1.2 indicates radiosensitization

Supplemental Table 7: *p* values for key pairwise comparisons in mouse tumor growth analysis

Compared Animal Cohorts		*p* value
Cohort#1	Cohort#2	
Control-NT	Control-B	0.7628
Control-NT	Control-X	0.0093
Control-NT	Control-X+B	<0.001
Control-B	Control-X	0.0795
Control-B	Control-X+B	<0.001
Control-X	Control-X+B	0.1357
shCasp8-NT	shCasp8-B	<0.001
shCasp8-NT	shCasp8-X	<0.001
shCasp8-NT	shCasp8-X+B	<0.001
shCasp8-B	shCasp8-X	<0.001
shCasp8-B	shCasp8-X+B	<0.001
shCasp8-X	shCasp8-X+B	<0.001
Control-NT	shCasp8-NT	<0.001
Control-B	shCasp8-B	<0.001
Control-X	shCasp8-X	0.9714
Control-X+B	shCasp8-X+B	<0.001

Supplemental Table 8: *p* values for key pairwise comparisons in mouse survival analysis

Compared Animal Cohorts		*p* value
Cohort#1	Cohort#2	
Control-NT	Control-B	0.4345
Control-NT	Control-X	<0.001
Control-NT	Control-X+B	<0.001
Control-B	Control-X	<0.001
Control-B	Control-X+B	<0.001
Control-X	Control-X+B	0.0206
shCasp8-NT	shCasp8-B	<0.001
shCasp8-NT	shCasp8-X	<0.001
shCasp8-NT	shCasp8-X+B	<0.001
shCasp8-B	shCasp8-X	<0.001
shCasp8-B	shCasp8-X+B	<0.001
shCasp8-X	shCasp8-X+B	<0.001
Control-NT	shCasp8-NT	0.4846
Control-B	shCasp8-B	<0.001
Control-X	shCasp8-X	0.4747
Control-X+B	shCasp8-X+B	<0.001

Supplemental Table 9: Key tumor growth parameters assessed in the mouse study presented in Figure 6

Key tumor growth parameters calculated as part of tumor growth analysis in the in vivo study					
**Treatment Conditions**	**Tumor Doubling Times (TDT)**		**Absolute Growth Delay (AGD)**	**Normalized Growth Delay (NGD)**	**Radiation Enhancement Factor (REF)**
	Mean	SD			
MOC1 Control - NT	8.3	3.2			
MOC1 Control - B	11.5	2.1	3.2		
MOC1 Control - X	14.4	2.1	6.1		
MOC1 Control - X+B	14.6	1.6	6.3	3.1	0.5
MOC1 shCasp8 - NT	7.1	1.2			
MOC1 shCasp8 - B	18.4	1.8	11.3		
MOC1 shCasp8 - X	15.8	1.7	8.7		
MOC1 shCasp8 - X+B	38	7.8	30.9	19.6	2.3

NT: No treatment; **B:** Birinapant; **X:** Radiation.

Tumor doubling time (TDT): number of days required for the doubling of tumor volumes from the day of randomization (Day27)

Absolute growth delay (AGD) = (Mean TDT for each treatment group – Mean TDT for the NT group)

Normalized growth delay (NGD) = (AGD for the X+B group – AGD for the B group)

Radiation Enhancement Factor (REF) = (NGD for the X+B group / AGD for the X group)

REF>1.2 indicates enhancement of radiation sensitivity
